# Genetic determinants of skin ageing: a systematic review and meta-analysis of genome-wide association studies and candidate genes

**DOI:** 10.1186/s40101-025-00384-9

**Published:** 2025-02-08

**Authors:** Chloe Wong, Jun Yan Ng, Yang Yie Sio, Fook Tim Chew

**Affiliations:** 1 Department of Biological Sciences, Faculty of Science, National University of Singapore, Singapore, 117,543 Singapore; 2 Allergy and Molecular Immunology Laboratory, Lee Hiok Kwee Functional Genomics Laboratories, Block S2, Level 5, 14 Science Drive 4, Lower Kent Ridge Road, Singapore, 117,543 Singapore

**Keywords:** Skin aging, Skin ageing, Genome-wide association study, Candidate gene, Meta-analysis, Genetic, Functional enrichment, Skin colour, Wrinkling, Skin cancer

## Abstract

**Background:**

Skin ageing is influenced by complex genetic factors. Various phenotypes such as wrinkling, pigmentation changes, and skin cancers have been linked to specific genetic loci. However, the underlying genetic mechanisms and pathways remain poorly understood. This systematic review and meta-analysis aims to summarise the genetic loci found to be associated with skin ageing phenotypes by published genome-wide association studies (GWAS) and candidate gene studies. We also evaluated the overall association of loci via meta-analysis and identified the association patterns to explore potential biological pathways contributing to skin ageing. The Web of Science, Embase, and PubMed databases were searched on January 2024 using specific exclusion criteria (e.g., study of non-human subjects, focus on skin diseases, or treatments) to identify relevant articles. There did not appear to be any significant publication bias observed across the all phenotypes.

**Main body:**

A total of 48 studies were included, revealing 30 loci that were confirmed to be associated with skin ageing by multiple studies (e.g., *AFG3L1P*: odds ratio 1.133 95% confidence interval [1.044, 1.222]; *BPIFA3*: 1.859 [1.567, 2.151]; *CLPTML1*: 1.164 [1.0.99, 1.229]; *CPNE7*: 0.905 [0.852–0.958]; *DEF8*: 1.186 [1.042, 1.331]; *IRF4*: 1.260 [1.025, 1.495]; *MYO16*: 2.303 [1.697, 2.908]; *PRDM16*: 1.105 [1.084, 1.127]; *RORA*: 1.391 [1.206, 1.577]; *SPG7*: 0.922 [0.897, 0.947]; *SPON1*: 2.214 [1.204, 3.225]; *SPTLC1*: 1.464 [1.432, 1.495]; *TYR*: 1.175 [1.007, 1.343]). The lack of significance for many loci may be due to studies analysing different SNPs within the same locus, weakening the overall associations. Several loci were associated with specific phenotypic categories (e.g., skin colour related, skin cancer related, wrinkling and sagging related), suggesting shared biological pathways are involved in the pathogenesis of different skin ageing phenotypes. This pattern was also observed in several of the loci that do not have a significant overall association with skin ageing.

**Conclusion:**

Despite significant heterogeneity among the included studies and the use of subjective visual methods for phenotype assessment, our review highlights the critical role of fundamental biological processes, such as development and cellular organisation, in skin ageing. Future research that targets the same SNP across multiple populations could strengthen the association of additional loci with skin ageing. Further investigation into these underlying biological processes would significantly advance our understanding of the pathogenesis of skin ageing phenotypes.

**Supplementary Information:**

The online version contains supplementary material available at 10.1186/s40101-025-00384-9.

## Introduction

### Background

As ageing populations become increasingly common worldwide, there is a growing need to understand the histological and physiological effects of ageing and ways to counter them [1]. Since the skin is the largest organ of the body and the organ most exposed to the external environment [2], observing the effects of ageing on the skin can provide valuable insights into the broader ageing processes occurring throughout the entire body, including on a genetic level.

Skin ageing is a complex and multifaceted process that can be defined as any change in the appearance, structure, and function of the skin over time. This is characterised by a broad range of numerous phenotypes, such as wrinkling, pigmentation changes, and sagging. These phenotypes can vary not only in their location and severity but also in their expression across different sex and ethnicities [3].

Thus, given the diverse nature of skin ageing phenotypes, much of the existing literature in the field of skin ageing are limited in their ability to study a wide range of phenotypes and the numerous genetic factors that may be responsible for them. While many genome-wide association studies (GWASes) and candidate gene studies have sought to identify genetic loci associated with skin ageing, most have been constrained to investigating only one or a few phenotypes at a time.

### Aims of the review

The aim of this review is to summarise all the genetic factors associated with a wide range of skin ageing phenotypes that have been identified in published GWASes and candidate gene studies. We also performed a meta-analysis to assess the association of selected significant loci with skin ageing and to identify patterns in these associations, particularly in terms of loci and specific skin ageing phenotypes. The meta-analysis will include a subset of data from the Singapore/Malaysia Cross-Sectional Genetic Epidemiology Study (SMCGES). Through this work, we aim to illustrate the potential biological pathways through which these loci may contribute to various skin ageing phenotypes, offering a more holistic view of the genetic factors involved in skin ageing.

## Methods

### Literature search strategy

The literature search was conducted in accordance with the Preferred Reporting Items for Systematic Reviews and Meta-Analyses (PRISMA) abstract checklist (Additional file 1) and PRISMA guidelines (Additional file 2). A primary search was performed on the Embase, PubMed, and Web of Science databases in January 2024. Search results were restricted to full-text English journal articles published any time before and in 2024. The search term used for all the databases included ‘skin aging’ or ‘skin ageing’ in the title or abstract as well as ‘gene*’ and ‘SNP’ in all index fields. To ensure a comprehensive coverage of all GWAS articles, an additional primary search was performed on the same three databases. The second set of search terms included ‘skin aging’ or ‘skin ageing’ in the title or abstract and ‘GWAS’ and ‘genotype’ in all index fields. The full list of search terms is summarised in Table 1. Eligible articles from the primary search were determined using a pre-defined eligibility criterion. The initial screening of articles was based on the titles and abstracts to exclude studies that did not meet the eligibility criteria. Studies that passed the first round of screening were then subjected to a second round of screening, which involved a detailed review of the full-length journal article to confirm relevance and eligibility for inclusion in the review.

**Table 1:** Summary of search terms used to find loci associated with skin ageing

Database	Search term	Number of articles
Embase	(skin ageing OR skin aging) AND (gene*) AND (SNP), all time	433
PubMed		41
Web of Science		900
Embase	(skin ageing OR skin aging) AND (GWAS) AND (genotype), all time	342
PubMed		38
Web of Science		107

### Eligibility criteria

All full-text journal articles in English were screened by two separate reviewers independently. Articles eligible for inclusion examined the association of SNPs with skin ageing phenotypes in any human subject via non-invasive methods in a non-experimental observational study. Studies were excluded if they met at least one of the following criteria: study examined non-human subjects, study did not examine any specific SNP, article focused on cosmetic products or treatments, study focused on skin diseases and disorders (e.g., allergies, psoriasis, cancer) instead of skin ageing phenotypes, and articles were reviews or meta-analyses. After the two rounds of screening—first by title and abstract, followed by the full text, 48 studies were included for data extraction.

Begg’s funnel plots and Egger’s test were used to assess publication bias. Begg’s funnel plots were used for forest plots with $\geq$ 2 studies, while Egger’s test was used for forest plots with $\ge$ 3 studies to estimate the asymmetry of data. An Egger’s test *p*-value of $<$ 0.05 was considered as the potential existence of publication bias.

In addition to the studies included from the literature search, data obtained from our own GWAS (SMCGES) was also included as another study of interest for further statistical analysis. Detailed descriptions of the characteristics of the SMCGES participants can be found in Lim et al. (2022) [4], Wong et al. (2023) [5], and Wong et al. (2022) [6].

### Data extraction

For each study included, the following variables were extracted: author(s), year of publication, article title, sample size, nationality, ethnicity, details of skin ageing phenotype studied (definition, location), and information of SNP studied (SNP rsID, effect allele). If there was a mismatch in the stated sample size and the number of participants with available genetic data, the sample size was recorded based on the number of participants used in the statistical analysis. Quantitative data were extracted for each SNP, including the *p*-value, odds ratio (OR), and confidence intervals (CIs). In cases where the OR and CIs were not reported, the beta coefficient and standard error were extracted instead and subsequently converted to OR and CIs for consistency. All *p*-values were also noted, regardless of whether they were significant (≤ 1 × 10^−5^ for GWAS articles and ≤ 0.05 for candidate gene articles).

Skin ageing phenotypes from our own GWAS were self-reported by participants using validated photo-numeric scales and photographic illustrations sourced from medical textbooks [7] [8]. We have previously shown that investigator-assessed phenotypes were concordant with our self-reported phenotypes [9].

To ensure uniformity across studies, the coordinates of all SNPs were standardised to the GRCh37/hg19 assembly using the UCSC LiftOver tool. Both the rsIDs and genomic coordinates of each SNP were documented accordingly. To ensure accurate matching of SNPs to their closest loci, the UCSC Genome Browser was used to identify the gene(s) associated with each SNP. The genomic coordinates of all the SNPs were loaded onto a custom track and matched to the corresponding gene positions on the GRCh37/hg19 human assembly. For intergenic SNPs, the closest genes upstream and downstream of the reported SNP were recorded. In cases where a SNP was in a position of two or more overlapping genes, all associated genes were noted. If a SNP was located on an uncharacterised gene, the Ensembl Stable ID (ENSG) was noted.

### Statistical analysis

Only SNPs with significant *p*-values were then noted and analysed for the generation of forest plots (≤ 1 × 10^−5^ for GWAS articles and ≤ 0.05 for candidate gene articles). For each study of interest, the SNP with the strongest OR and 95% CI for each studied locus was selected for extraction. The specific skin ageing phenotype associated with the SNP in the respective study was also recorded for meta-analysis. Meta-analyses were conducted for each locus found to be associated with a skin ageing phenotype by at least two different studies. This analysis was conducted using the metafor package with R software Version 4.3.1 in RStudio Version 2023.06.1+524." The latest version of RStudio is 2024.12.0+467 as of 30 December 2024. The metafor package is a comprehensive tool for conducting meta-analyses within the R environment, capable of generating forest plots. These forest plots consolidates the results of individual studies and the overall combined estimate into a single plot. Forest plots were generated for each risk factor (i.e., each locus). For each locus, the weights of each study were calculated via the metafor package by inversing the standard deviation. To refine the analyses, studies contributing a weight of less than 1% were excluded as their contributions to the overall findings were negligible. Forest plots were then generated according to the data for each locus.

An overall OR and CI was calculated for each forest plot. A locus was considered to have overall significant association with skin ageing if the pooled estimate did not cross the line of no effect (odds ratio = 1). If the pooled estimate crosses the line of no effect, the locus was considered to not have an overall significant association with skin ageing. The inconsistency index (*I* ^2^) was calculated to assess the heterogeneity in the pooled risk estimate. An *I* ^2^ value ≥ 50% and *p*-value < 0.05 were considered statistically significant for heterogeneity.

### Functional enrichment

Functional enrichment was carried out using the functional profiling feature (g:GOst) on g:Profiler. A stepwise approach with exclusion was employed to systematically refine the set of 307 non-intergenic loci. Functional enrichment was conducted on the entire set of loci from multiple databases—Gene Ontology (GO), biological pathways (KEGG, Reactome, WikiPathways), regulatory motifs in DNA (TRANSFAC (TF), miRTarBase), protein databases (Human Protein Atlas, CORUM), and Human Phenotype Ontology (HP). The stepwise approach was conducted as follows: Genes that were categorised under the most prevalent overarching functional category were removed from the set. The remaining loci underwent another round of functional enrichment. This iterative process continued until the remaining loci could no longer be classified under any functional category. Under this refinement process, genes associated with pigmentation were removed first, followed by those related to development. Finally, genes involved in cytoplasmic and cytoskeletal functions were excluded. The remaining genes that could not be classified into any functional category were grouped under ‘others’. To explore whether the sequence in which categories were removed affected the downstream enrichment process, overarching categories were removed in different orders (e.g., loci related to development were removed first before loci associated with pigmentation were removed). We found that the same three categories reported above arise regardless of the order in which they were removed. The full set of results from functional enrichment was retrieved and reported in Additional File 3.

## Results and discussion

### Overview of data analysis from literature search

**Fig. 1:**
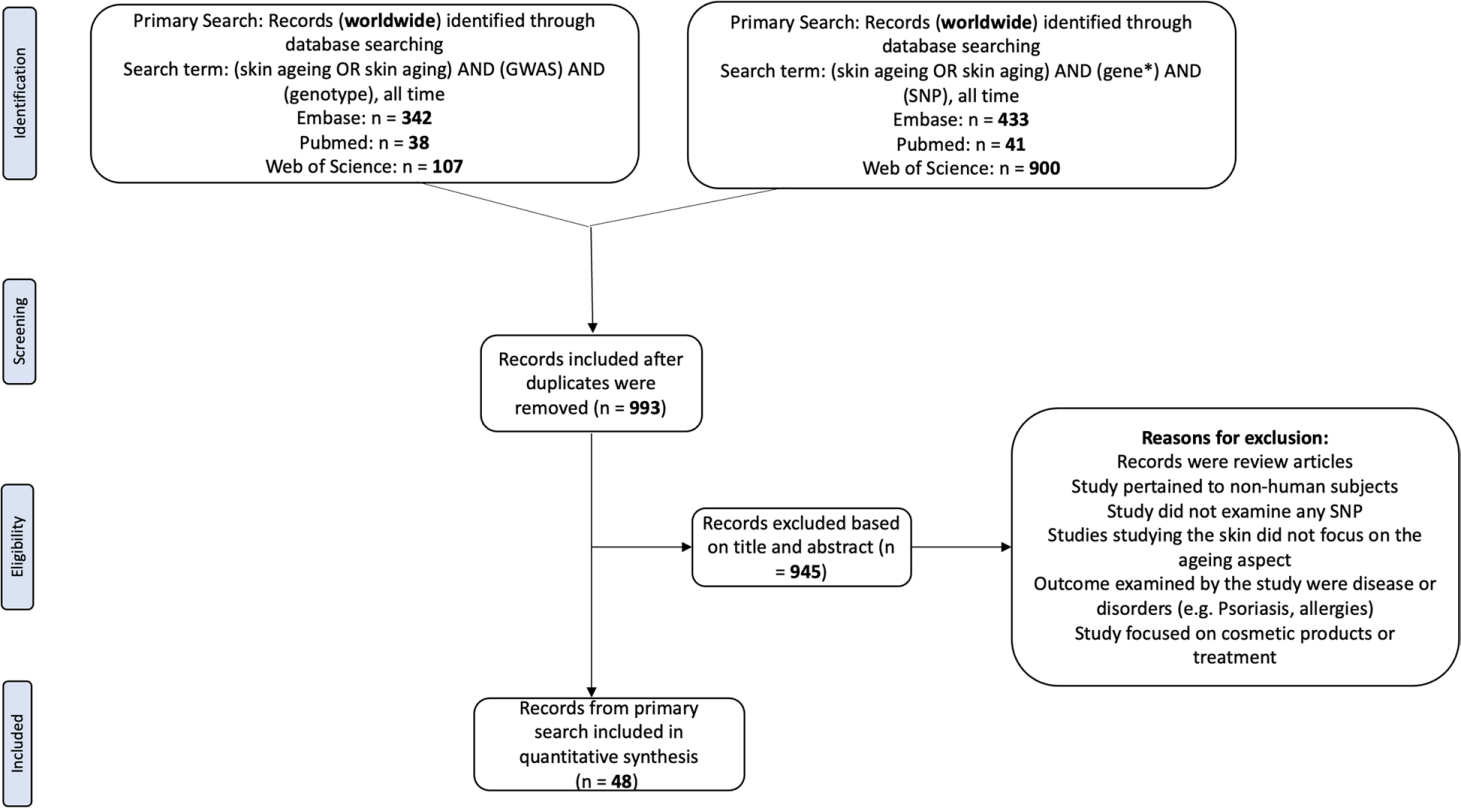
Workflow for screening through articles to include in the review in accordance with PRISMA guidelines

In addition to the exclusion criterion mentioned, articles that did study the effect of a candidate gene on skin ageing but did not focus on a specific skin ageing phenotype were not included [10]. Following the screening process, 48 eligible studies were shortlisted for analysis (Fig. 1). Given that Begg’s funnel plots were mostly symmetrical, and Egger’s test *p*-values were mostly > 0.05, there did not appear to be any significant publication bias observed across the all phenotypes.

From these studies, we consolidated a total of 2408 SNPs and 3844 SNP-phenotype associations with skin ageing phenotypes. This was based on the cut-off value of the *p*-values depending on the type of study (SNPs from GWAS studies with a *p*-value < 1 × 10^−5^ and SNPs from candidate gene studies with a *p*-value < 0.05). These SNPs were located in 410 non-intergenic and intergenic loci. Since these SNPs were associated with skin ageing, the 410 loci were termed skin ageing loci.

### Overview of study characteristics

The selected studies exhibited a wide variety of characteristics. Among the studies, 39 were GWASes and 9 were candidate gene studies. The study populations originated from 16 countries, spanning Europe, America, and Asia. Sample sizes varied significantly, with the smallest study having 39 participants [11] and the largest comprising 287,137 participants [12]. While some studies focused on participants of only one sex, the majority included male and female participants. Participants ranged from 18 [13] [14] to 98 years old [15], with the narrowest age range reported being 54–55 years [16] and the widest being 18 to 79 years [13]. The characteristics of each included study were retrieved and summarised in Additional File 4.

### Overview of meta-analysis

After the adjustment for studies that contributed a weightage of less than 1%, 95 loci were reported as significantly associated with skin ageing by more than 1 paper and were followed up on by other studies, of which there were 198 unique SNPs located across these 95 loci (Additional File 5). Meta-analyses showed that out of the 95 loci, 30 loci have been confirmed to be significantly associated with skin ageing by multiple studies. The forest plots of these loci were presented in Additional Files 6, 7, 8, 9, 10, 11, 12, 13, 14, 15, 16, 17, 18, 19, 20, 21, 22, 23, 24, 25, 26, 27, 28, 29, 30, 31, 32, 33, 34, and 35. The overall association of the other 65 loci with skin ageing was found to not be significant.

### Overview of skin ageing phenotypes in meta-analysis

The meta-analyses included a range of skin ageing phenotypes—wrinkling, solar elastosis, pigmented spots, perceived skin darkness, inability to tan, freckles, Favre-Racouchot syndrome, cutaneous squamous cell carcinoma, basal cell carcinoma, cutaneous melanoma, non-facial solar lentigines, increased perceived age, upper lip fullness, Forehead Wrinkles, sagging eyelids, lower lip fullness, Glabellar Frown Wrinkles, Melomental Folds, and lower skin reflectance.

A systematic review by Ng and Chew (2022) [3] previously grouped 56 different skin ageing phenotypes into four categories: (1) skin wrinkling and sagging-related phenotypes, (2) skin colour-related phenotypes, (3) skin cancer-related phenotypes, and (4) skin global impression phenotypes.

Skin cancer-related phenotypes are classified as skin ageing phenotypes due to their close association with skin colour-related pathways, suggesting they may share common genetic factors. For instance, solar lentigines—a type of skin colour-related phenotype—can serve as precursors to melanoma, a skin cancer originating in melanocytes [17]. Similarly, actinic keratosis, another pigmentation-related phenotype, has a small risk of progression to squamous cell carcinoma [18]. Furthermore, increased Sun and UV exposure is a well-documented risk factor for both skin colour-related changes and skin cancers, underscoring the likelihood of shared genetic pathways [19] [20]. This connection positions skin cancer-related phenotypes as an integral component of skin ageing indicators.

It is noteworthy that many skin ageing phenotypes associated with the same genetic locus tend to be similar in nature, often falling into the same skin ageing phenotype category. In some cases, the same phenotype is identified by different terms across studies (e.g., perceived skin darkness and lower skin reflectance). For instance, the *SPTLC1* gene has been associated with wrinkling in two separate studies. Similarly, the phenotypes associated with *RORA*, *MYO16*, and *PRDM16* fall under the category of skin wrinkling or sagging-related phenotypes, while the majority of phenotypes linked to *AFG3L1P* were related to skin colour.

The fact that certain loci were consistently associated with skin ageing phenotypes within specific categories suggests that despite variations in the specific phenotypes identified across studies, the biological pathways influenced by these loci may be inclined toward particular patterns of skin ageing. In the next section, we will discuss the loci that display a tendency to be associated with skin ageing phenotypes of a similar nature or category.

### Loci associated with skin colour-related phenotypes

#### AFG3L1P

Five studies were included in the meta-analysis for *AFG3L1P* (*OR* = 1.133, *CI* = 1.044–1.222) (Additional File 6). Four of these studies identified *AFG3L1P* as being associated with skin colour-related phenotypes, such as facial pigmented spots, perceived skin darkness, and inability to tan. The fifth study reported an association with a skin wrinkling or sagging phenotype, specifically solar lentigines.

**Fig. 2:**
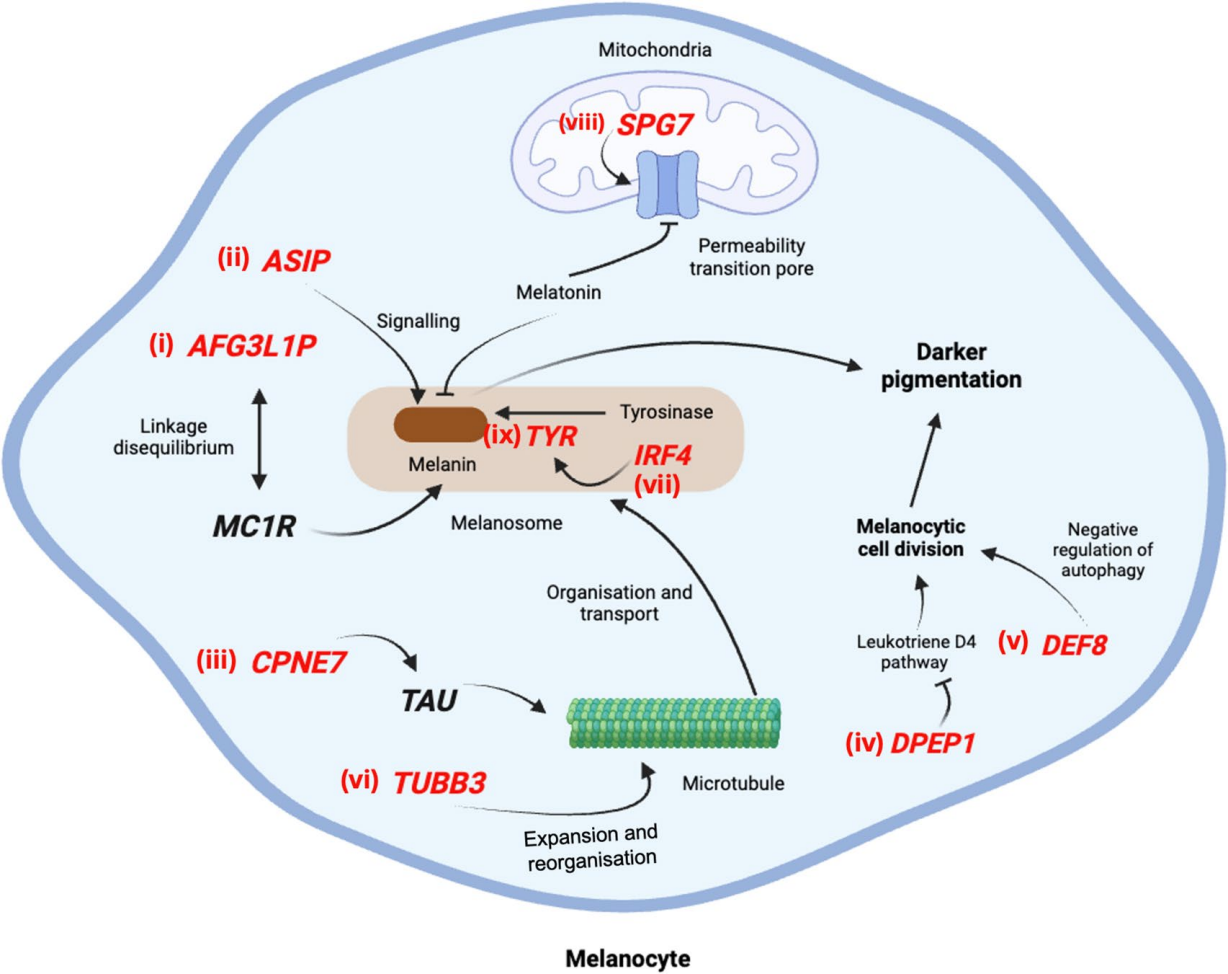
Summary of the potential pathways and mechanisms of genes associated with skin colour-related phenotypes. (i) *AFG3L1P* is in strong linkage disequilibrium with *MC1R*, a gene critical for melanin synthesis; (ii) *ASIP* promotes the synthesis of darker eumelanin over lighter pheomelanin; (iii) *CPNE7* and (iv) *TUBB3* contribute to melanosome organisation through its role in microtubule expansion; (v) *DPEP1* and (vi) *DEF8* inhibit melanocyte cell division via the negative regulation of the leukotriene D4 signalling pathway and autophagy, respectively; (vii) *IRF4* upregulates *TYR* expression, a gene essential for melanin synthesis; (viii) *SPG7* levels inversely correlate with melatonin, which negatively regulates melanin synthesis, linking *SPG7* to melanin production; and (ix) *TYR* catalyses the conversion of tyrosine to melanin

Marley (2020) [21] identified several SNPs within the pseudogene, *AFG3L1P* that exhibit strong linkage disequilibrium with an *MC1R* SNP, a key gene involved in pigmentation. Given that elevated *MC1R* expression promotes increased eumelanin synthesis, leading to darker pigmentation [22], we hypothesise that the association between *AFG3L1P* and skin colour-related phenotypes may be driven by its linkage disequilibrium with *MC1R* (Fig. 2i). However, research by Bánfai et al. [23] suggests that *AFG3L1P* may be one of the rare exceptions among pseudogenes that were actually translated, indicating a potential protein-coding role that could contribute to skin colour-related phenotypes. Further studies are needed to fully elucidate the impact of the protein-coding function of *AFG3L1P*.

## Intergenic region between *ASIP* and *ENSG00000287853*

Two studies identified the intergenic region between *ASIP* and *ENSG00000287853* as a locus associated with skin ageing (*OR* = 1.391, *CI* = 1.213–1.568) (Additional File 7). The associated phenotypes—inability to tan and freckles—fall under the category of skin colour-related traits. Kanetsky et al. (2002) [24] found that reduced *ASIP* expression could affect pigmentation signalling, resulting in higher eumelanin production relative to pheomelanin (Fig. 2ii). Notably, the same SNP, rs4911414, identified in our meta-analysis as linked to these skin colour-related phenotypes, was also associated with Sun sensitivity and freckling in the study by Yardman-Frank and Fisher (2021) [25]. The involvement of *ASIP* in pigmentation pathways, combined with the consistent association of rs4911414 with skin ageing phenotypes across studies, further strengthens the link between *ASIP* and skin colour-related ageing traits.

## CPNE7

*CPNE7* was found to be associated (*OR* = 0.905, *CI* = 0.852––0.958) with facial pigmented spots and the inability to tan, both of which are classified as skin pigmentation-related phenotypes (Additional File 8). *CPNE7* is believed to play a role in intracellular processes related to cytoskeleton organisation [26].

Bai et al. (2022) found that *CPNE7* was able to induce microtubule expansion and reorganisation via regulating the expression of tubulin-associated unit (*TAU*), a microtubule-associated protein [27]. The importance of microtubules and the cytoskeleton in the movement and organisation of melanosomes [28] points to a potential connection between CPNE7 and pigmentation-related processes **(**Fig. 2iii**)**.

## Intergenic region between *CPNE7* and *DPEP1*

Of the three studies that identified the intergenic region between *CPNE7* and *DPEP1* as being associated with skin ageing (*OR* = 1.493, *CI* = 1.130–1.856), two linked this locus to skin colour-related phenotypes, specifically inability to tan and freckles (Additional File 9). *DPEP1* encodes a zinc-binding metalloproteinase involved in leukotriene metabolism, antibiotic processing, and dipeptide hydrolysis [29].

 The inhibition of the leukotriene D4 signalling pathway by *DPEP1* [30], which has been found to induce cell division in melanocytes [31], could account for its association with skin colour-related phenotypes (Fig. 2iv).

## DEF8

Five studies identified *DEF8* as being associated with various skin ageing phenotypes (*OR* = 1.186, *CI* = 1.042–1.331) (Additional File 10). These include two phenotypes related to skin wrinkling and sagging (solar lentigines) and skin cancer (cutaneous squamous cell carcinoma) categories. The remaining three phenotypes fall under the skin pigmentation-related category, including perceived skin darkness, facial pigmented spots, and the inability to tan. *DEF8* is involved in the regulation of lysosome positioning and distribution, as well as bone resorption [32]. Notably, the SNP rs4268748, associated with cutaneous squamous cell carcinoma in our meta-analysis, has also been found to be linked to the expression levels of *CDK10*, a gene crucial for cell cycle progression in the GTEx database, although the exact mechanism by which it does is not known [33].

*DEF8* is believed to be part of the Rubicon protein family, known for its role in the negative regulation of autophagy [34]. A reduced autophagic rate, particularly in melanocytes, could lead to decreased degradation of tyrosinase, thereby increasing melanin synthesis and pigmentation [35]. Consequently, the role of *DEF8* in regulating lysosomes and autophagy may link it to skin colour-related phenotypes (Fig. 2v).

## Intergenic region between *DEF8 *and *TUBB3*

Both studies included in the meta-analysis identified the same SNP in the intergenic region between *DEF8* and *TUBB3* as being associated with skin pigmentation-related phenotypes, specifically pigmentation spots and perceived skin darkness (*OR* = 0.937, *CI* = 0.921–0.953) (Additional File 11). This finding reinforces the association of the *DEF8* locus with pigmentation-related aspects of skin ageing. *TUBB3* encodes a critical component of microtubules, which play a vital role in cellular processes such as intracellular transport [36] and cell division [37].

Combining the fact that microtubules are implicated in melanosome transport [38] and that *TUBB3* expression has been shown to be reduced in senescent melanocytes [39], this provides a direct possible link between *TUBB3* and pigmentation-related phenotypes (Fig. 2vi).

This intergenic region between *DEF8* and *TUBB3* contains two promoters for the *DEF8* gene, suggesting that SNPs in this region may influence the expression of *DEF8* [40]. This finding is consistent with the association of both *DEF8* and this intergenic region with skin colour-related phenotypes.

## ENSG00000259006

Of the three studies included in the meta-analysis for *ENSG00000259006*, two found the gene to be associated with skin colour-related phenotypes—facial pigmented spots and freckles (*OR* = 4.874, *CI* = 2.040–7.708) (Additional File 12). Its function as a long non-coding RNA remains unknown and more research is required to uncover its relation to skin colour-related phenotypes (Additional File 20).

## ENSG00000286364

Four studies were included in the meta-analysis for *ENSG00000286364* (*OR* = 1.327, *CI* = 1.135–1.519) (Additional File 13). Three of these studies found an association between *ENSG00000286364* and skin pigmentation-related phenotypes, including the inability to tan and freckles. The fourth study identified a link with basal cell carcinoma. *ENSG00000286364* is a long non-coding RNA and its biological function remains largely unknown, particularly in relation to skin pigmentation-related phenotypes. Further investigation is needed to elucidate the mechanisms by which this gene influences these skin ageing traits.

## IRF4

Our meta-analysis identified seven studies that found *IRF4* to be associated with skin ageing (*OR* = 1.260, *CI* = 1.025–1.495), with most linking the same SNP, rs12203592, on the gene to pigmentation-related phenotypes such as perceived skin darkness, inability to tan, and pigmentation spots (Additional File 14). *IRF4* encodes a transcription factor that regulates immune responses, particularly in the differentiation of CD8( +) dendritic cells [41] and has also been implicated in pigmentation traits, including skin, hair, and eye colour [42].

Chhabra et al. (2017) [43] suggested that increased expression levels of *IRF4* may lead to elevated expression of *TYR*, a key gene in melanin production (Fig. 2vii). This regulatory relationship could explain the association between *IRF4* and skin pigmentation-related phenotypes.

## SPG7

*SPG7* was found to be associated with facial pigmented spots and perceived skin darkness (*OR* = 0.922, *CI* = 0.897–0.947), both of which are categorised as skin pigmentation-related phenotypes (Additional File 15). *SPG7* encodes a protease involved in maintaining the proteostasis of inner mitochondrial membrane proteins and plays a key role in forming and regulating the mitochondrial permeability transition pore [44].

Melatonin has been found to inhibit the mitochondria transition pore via the maintenance of the mitochondrial membrane potential [45], and thus, *SPG7*, as a critical component of the mitochondrial permeability transition pore [46], may be associated with the expression of melatonin. Since melatonin has also been shown to potentially inhibit melanogenesis (i.e., the production of melanin) [47], *SPG7* may also be associated with skin colour-related phenotypes via its association with melatonin. Its role in controlling mitochondrial function suggests a connection to the regulation of pigmentation processes through the influence of melatonin on melanin production (Fig. 2viii).

## TYR

Six studies found *TYR* to be associated with skin ageing phenotypes (*OR* = 1.175, *CI* = 1.007–1.343), with four studies specifically reporting its association with skin pigmentation-related phenotypes, such as inability to tan, lower skin reflectance, and freckles (Additional File 16). The *TYR* gene encodes an enzyme critical for the conversion of tyrosine to melanin [48], the pigment responsible for skin, hair, and eye colouration (Fig. 2ix). Consequently, *TYR* plays a fundamental role in regulating skin pigmentation, which directly links it to pigmentation-related skin ageing phenotypes.

## Loci associated with wrinkling- and sagging-related phenotypes

### BCAR3

*BCAR3* was identified as being associated with skin ageing in two studies, including our own GWAS (*OR* = 2.966, *CI* = 1.301–4.631) (Additional File 17). Both phenotypes linked to this locus—wrinkling and Favre-Racouchot syndrome—fall within the category of skin wrinkling and sagging. *BCAR3* encodes a protein that plays a critical role in various cell signalling pathways related to cell proliferation, migration, and extracellular matrix assembly [49].

**Fig. 3:**
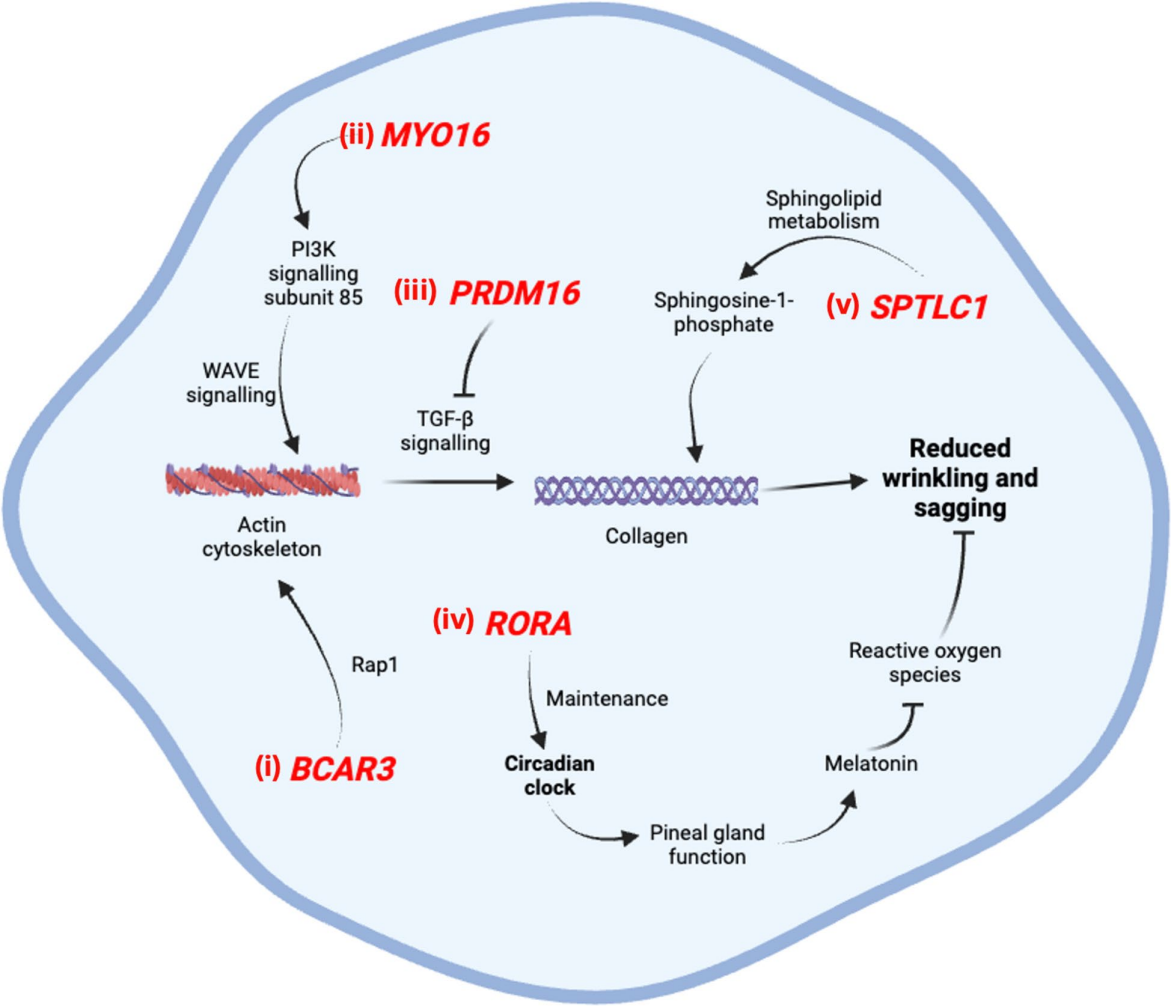
Summary of the potential pathways and mechanisms of genes associated with wrinkling and sagging-related phenotypes. (i) *BCAR3* and (ii) *MYO16* promote actin polymerisation through distinct signalling pathways, leading to collagen production—a key factor in wrinkle reduction; (iii) *PRDM16* inhibits the TGF-β signalling pathway, which activates collagen synthesis, leading to reduced wrinkling and sagging; (iv) *RORA* modulates pineal gland function, promoting melatonin production, which reduces reactive oxygen species and contributes to wrinkle reduction; and (v) *SPTLC1* is involved in sphingolipid metabolism, generating a metabolite that stimulate collagen synthesis

*BCAR3* is believed to play a key role in actin cytoskeleton assembly through the activation of the GTPase Rap1 [50], [51]. Given that the TGF-β pathway links the actin cytoskeleton to collagen production [52]—an essential process in minimising wrinkles—*BCAR3* likely contributes indirectly to wrinkle formation via downstream signalling pathways. Its involvement in cytoskeletal organisation and collagen synthesis underscores its potential role in skin ageing and wrinkle reduction (Fig. 3i).

## ENSG00000233635

Both studies that were included in the meta-analysis identified *ENSG00000233635* as being associated with general wrinkling (*OR* = 0.827, *CI* = 0.826–0.828) (Additional File 18). The gene is a long non-coding RNA, which function is still unclear and further investigation is required to deduce its relation to the pathogenesis of wrinkling in the skin.

## ENSG00000242593

Two studies, including our own GWAS, identified *ENSG00000242593* as being associated with wrinkling- and sagging-related phenotypes, namely, sagging eyelids and upper lip fullness (*OR* = 0.689, *CI* = 0.451–0.927) (Additional File 19). *ENSG00000242593* falls under the class of long non-coding RNA and more research is required to discover the underlying mechanisms of the gene that contribute to wrinkling- and sagging-related phenotypes.

## MYO16

*MYO16* was identified as being associated with skin ageing in two studies (*OR* = 2.303, *CI* = 1.697–2.908), both of which linked the gene to wrinkling- and sagging-related phenotypes, specifically general wrinkling and Glabellar Frown Wrinkles (Additional File 20). *MYO16* encodes a motor protein that is thought to be involved in signalling pathways which regulates cell cycle progression [53].

*MYO16* interacts with the PI3-kinase (PI3K) regulatory subunit p85, linking it to the activation of the WASp-family verprolin homologous protein (WAVE) signalling pathway, which regulates actin polymerisation [54]. Actin polymerisation, in turn, triggers the TGF-β pathway responsible for collagen production [55]. This connection suggests that *MYO16* plays a role in the indirect pathway of collagen synthesis, explaining its association with wrinkling (Fig. 3ii).

## PRDM16

*PRDM16* was identified as being associated with general wrinkling in both studies included in our meta-analysis (*OR* = 1.105, *CI* = 1.084–1.127) (Additional File 21). This gene encodes a transcription factor that plays a key role in the differentiation of brown adipose tissue and the regulation of transcription and signalling pathways [56]. This includes the blocking of the TGF-β signalling pathway [57]—the pathway that activates collagen production [55], which is a crucial element of preventing wrinkling (Fig. 3iii).

## RORA

Our meta-analysis found *RORA* to be associated with two skin ageing phenotypes: sagging eyelids and Melomental Folds (*OR* = 1.391, *CI* = 1.206–1.577), both of which are categorised as wrinkling or sagging-related phenotypes (Additional File 22). *RORA* encodes a nuclear receptor involved in the regulation of embryonic development [58], immune responses [59], circadian rhythm [60], and the metabolism of various compounds [61].

*RORA* is essential for maintaining the circadian clock [62], which regulates pineal gland function and enhances melatonin secretion [63]. Melatonin, known for its ability to reduce reactive oxygen species (ROS), plays a key role in mitigating wrinkle formation [64]. Therefore, the involvement of *RORA* in circadian regulation and melatonin production likely explains its association with wrinkling and sagging-related phenotypes (Fig. 3iv).

## SPTLC1

Both studies in our meta-analysis found *SPTLC1* to be associated with general wrinkling (*OR* = 1.464, *CI* = 1.432–1.495) (Additional File 23). *SPTLC1* encodes an enzyme crucial for sphingolipid biosynthesis, which plays a vital role in various cellular processes, including structural components, modulation of cellular responses, and inflammatory responses [65]. The importance of sphingolipid biosynthesis to maintain a healthy skin barrier and skin moisture is reinforced by the findings of Kim et al. (2021) [66].

Sphingosine-1-phosphate, a key metabolite in sphingolipid metabolism, has been shown to promote collagen synthesis [67]—an essential factor in maintaining skin structure and reducing wrinkles. Thus, *SPTLC1* plays a central role in sphingolipid metabolism, which links it to skin wrinkling phenotypes (Fig. 3v).

## Loci associated with skin cancer-related phenotypes

### Intergenic region between *BNC2 *and *ENSG00000237153*

**Fig. 4:**
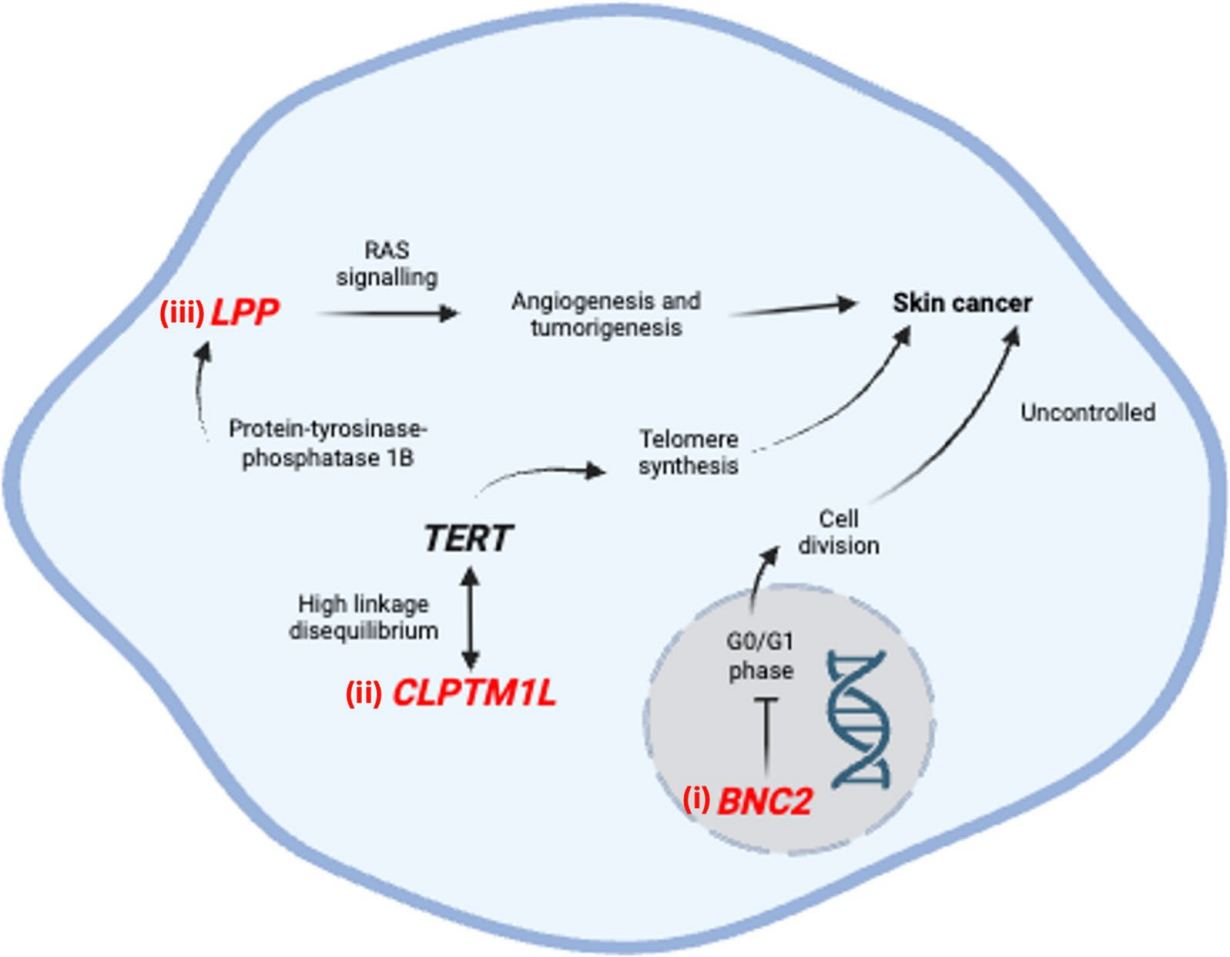
Summary of the potential pathways and mechanisms of genes associated with skin cancer-related phenotypes. (i) *BNC2* induces G0/G1 phase arrest, preventing uncontrolled cell division and thereby reducing skin cancer risk; (ii) *CLPTM1L* is in high linkage disequilibrium with *TERT*, a gene essential for telomere synthesis and linked to increased skin cancer susceptibility; (iii) *LPP* promotes angiogenesis and tumourigenesis through activation of the *Ras* signalling pathway

Three studies identified an association between the intergenic region between *BNC2* and *ENSG00000237153* (*OR* = 0.907, *CI* = 0.894–0.920) and skin ageing phenotypes specifically related to skin cancer, including basal cell carcinoma and cutaneous squamous cell carcinoma (Additional File 24). *BNC2* encodes a zinc finger protein that functions as a transcription factor [68] and plays a role in skin pigmentation [69]. Liu et al. (2022) [70] has found that *BNC2* could inhibit the cell growth, migration, and invasion of ovarian cancer cells by inducing G0/G1 phase arrest, suggesting its potential role as a tumour suppressor (Fig. 4i). Of particular interest is the SNP rs12350739, identified in from the meta-analysis as associated with cutaneous squamous cell carcinoma [71]. This SNP is located in an enhancer region that regulates *BNC2* transcription in melanocytes, suggesting a potential link between *BNC2* and skin cancer-related phenotypes.

## CLPTM1L

Of the three studies that identified *CLPTM1L* as being associated with skin ageing, two linked the gene to basal cell carcinoma, a skin cancer-related phenotype (*OR* = 1.164, *CI* = 1.099–1.229) (Additional File 25). *CLPTM1L* is predicted to encode a transmembrane protein and has been found to be upregulated in cisplatin-resistant cancer cells. It also shows strong linkage disequilibrium with *TERT*, a gene critical for telomere synthesis [72]. Given that telomere length has been associated with an increased risk of basal cell carcinoma [73], the linkage disequilibrium between *CLPTM1L* and *TERT* may explain the observed association between *CLPTM1L* and skin cancer-related phenotypes (Fig. 4ii).

## Intergenic region between *ENSG00000271399* and *ENSG00000271475*

Our meta-analysis identified an association between the intergenic region between *ENSG00000271399* and *ENSG00000271475* and basal cell carcinoma and cutaneous squamous cell carcinoma (*OR* = 1.235, *CI* = 1.067–1.402), both of which are categorised as skin cancer-related phenotypes (Additional File 26). Both *ENSG00000271399* and *ENSG00000271475* are pseudogenes and further research is needed to clarify their relevance to the pathogenesis of these forms of skin cancer.

## LPP

Both studies included in the meta-analysis for *LPP* found the gene to be associated with skin cancer-related phenotypes, specifically basal cell carcinoma and cutaneous squamous cell carcinoma (*OR* = 1.114, *CI* = 1.107–1.121) (Additional File 27). *LPP* encodes a protein that plays a dual role in cellular structure and signalling, contributing to cell adhesion, motility, and permeability [74].

*LPP* has been identified as a substrate of protein-tyrosine-phosphatase 1B, an enzyme involved in the Ras signalling pathway [75]. The Ras pathway stimulates angiogenesis and regulates various cellular functions linked to tumorigenesis [76]. Therefore, the involvement of *LPP* in this signalling pathway may suggest a potential connection to skin cancer-related phenotypes (Fig. 4iii).

Notably, several genes exhibiting a tendency to be associated with skin colour-related phenotypes were also reported in one or two studies to be linked to skin cancer phenotypes. Examples include *DEF8* (Additional File 10), *IRF4* (Additional File 14), and *TYR* (Additional File 16). Additionally, a few genes that did not show a clear association with any specific skin ageing phenotype were found by separate studies to be related to both skin colour and skin cancer phenotypes. These include genes such as *BPIFA3* (Additional File 28), *ENSG00000286803* (Additional File 13), and *NCOA6* (Additional File 33) which all had two studies that found the genes to be associated with one skin colour-related phenotype (freckles) and one skin cancer-related phenotype (cutaneous melanoma).

This observed pattern suggests that genes associated with skin colour-related traits may also play a role in skin cancer phenotypes, supporting the hypothesis of shared genetic factors between skin colour and skin cancer phenotypes.

## Loci with nonsignificant associations

While our meta-analysis identified 65 loci that were not significantly associated with skin ageing, we observed a recurring pattern among these loci: skin ageing phenotypes linked to a given locus often belonged to the same category. For instance, the phenotypes associated with *ANKRD11* (*OR* = 0.969, *CI* = 0.807–1.132) (Additional File 36), *CDK10* (*OR* = 1.230, *CI* = 0.908–1.552) (Additional File 37), *DBNDD1* (*OR* = 0.930, *CI* = 0.772–1.089) (Additional File 38), and *GAS8* (*OR* = 0.918, *CI* = 0.725–1.112) (Additional File 39) were all related to skin colour. Similarly, both phenotypes associated with *FAR2* (*OR* = 1.354, *CI* = 0.027–2.681) (Additional File 40) and *TANC2* (*OR* = 1.018, *CI* = 0.643–1.393) (Additional File 41) involved wrinkling and sagging.

The lack of overall significance for many of these loci may be due to different studies analysing different SNPs within the same locus, which could have varying effects on the same skin ageing phenotype, diluting the overall association. As we observed that skin ageing phenotypes linked to a given locus often belonged to the same category, we speculate that by studying the same SNP across multiple populations, future work could strengthen the overall association between some of these loci and skin ageing.

### Overview of functional enrichment

**Fig. 5:**
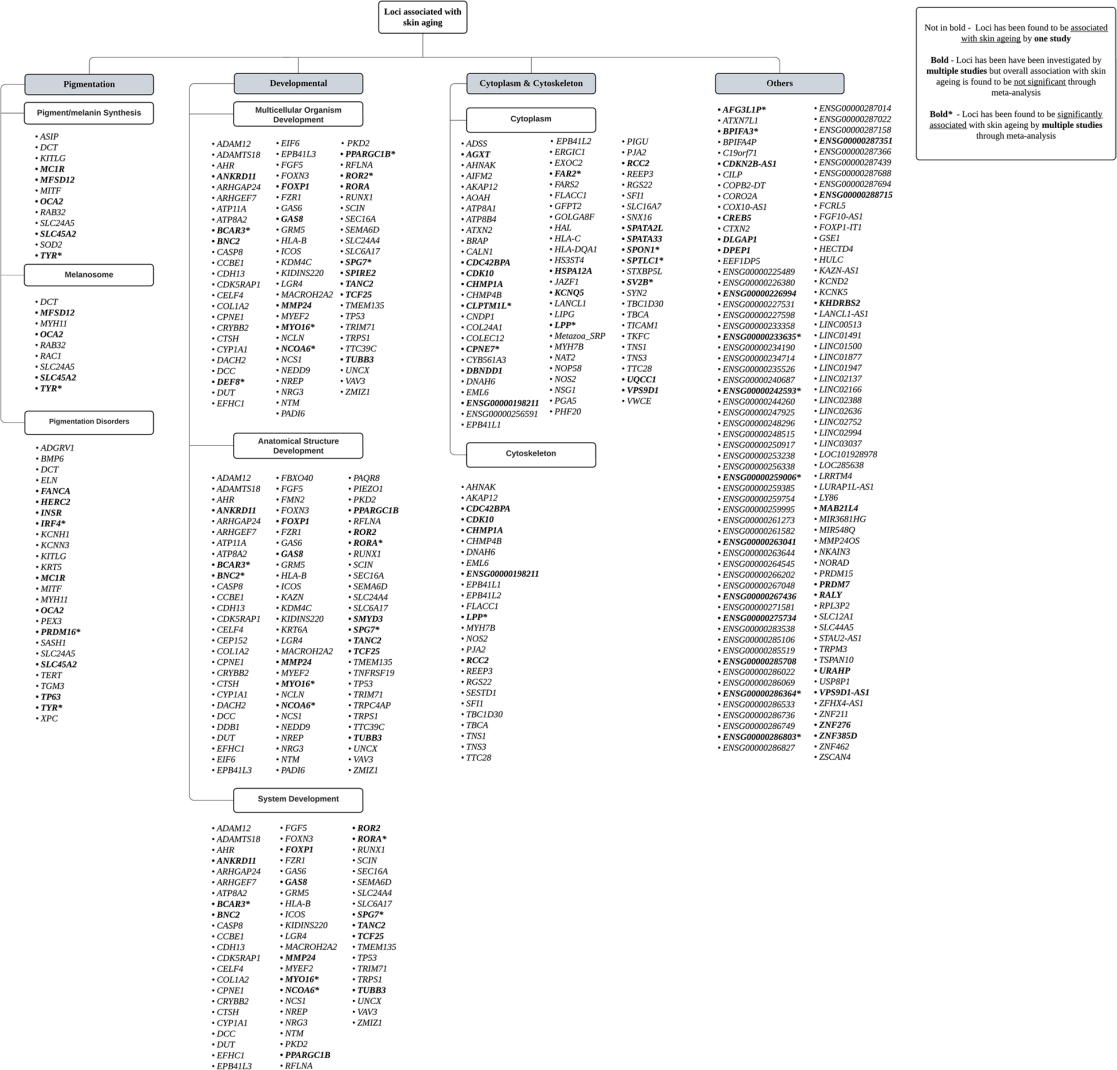
Flowchart depicting the classification of genes associated with skin ageing phenotypes, as identified by at least one study, into distinct categories following functional enrichment analysis

Functional enrichment analysis of the identified loci revealed four main categories that play crucial roles in skin ageing: (1) Pigmentation, (2) development, (3) cytoplasm and cytoskeleton, and (4) others (Additional File 3). Each of these categories was further divided into subcategories, reflecting the specific biological processes influenced by the associated genes (Fig. 5).

The ‘pigmentation’ category encompassed genes (e.g., *ASIP* and *MC1R*) integral to pigment and melanin synthesis, which play a crucial role in determining skin tone and mediating the response of the skin to UV exposure [77]. This category also included genes associated with the structure and function of melanosomes—the cellular organelles within melanocytes responsible for the production, storage, and transport of pigment [78]—such as *MFSD12*, *OCA2*, and *TYR*. Furthermore, genes implicated in pigmentation disorders, including *FANCA*, *HERC2*, and *PRDM16*, were linked to conditions like hypopigmentation and hyperpigmentation and were also classified under this category (Fig. 5).

The ‘development’ category includes genes essential for the progression of a multicellular organism from a zygote or young adult to a fully developed adult and ultimately to an aged individual. Notable genes in this category include *DEF8*, *SPIRE2*, and *RORA*. This category also encompasses genes involved in the formation and development of anatomical structures, such as *BCAR3* and *SMYD3*. Additionally, genes critical to the maturation and functioning of biological systems, including *BNC2*, *PPARGC1B*, and *SPG7*, are classified under this category. These genes collectively contribute to the complex processes of growth, development, and ageing (Fig. 5). The ‘cytoplasm and cytoskeleton’ category comprises genes that regulate cytoplasmic activities and cytoskeleton organisation. In the subcategory of ‘cytoplasm,’ genes such as *CLPTM1L* and *SPTLC1* play key roles in processes like regulation of apoptosis [10], building structural components, and the regulation of various reactions [65]. Meanwhile, genes like *CDC42BPA* and *LPP* are crucial for cytoskeleton organisation, which is essential for maintaining cell shape and supporting cellular activities such as movement, division, and endocytosis [79] (Fig. 5). These genes ensure the structural integrity and dynamic functions of cells, both of which are critical to overall cellular health and longevity.

Genes confirmed to be significantly associated with skin ageing across multiple studies are distributed across various categories and subcategories, including some that do not fit into a specific category (Fig. 5). Examples include *RALY*, which is involved in the splicing and metabolism of mRNA (80), *BPIFA3*, which likely plays a role in immune response, in terms of pathogen recognition [81], and several ENSG genes with functions that have yet to be determined.

Our functional enrichment analysis revealed that the majority of genes associated with skin ageing phenotypes are categorised under broad but essential biological processes, such as development and cytoplasm and cytoskeleton organisation. This suggests that the biological pathways contributing to the pathogenesis of skin ageing phenotypes are fundamental processes integral to the daily functioning and maintenance of cells.

Genes related to pigmentation are crucial in offering protection of the skin against UV damage by regulating melanin production and skin tone, reducing genetic damage and risks of cancer. Genes under the ‘development’ category ensure the proper growth and functioning of the human body by facilitating the formation of complex anatomical structures and biological systems, enabling the development of an adaptable organism. Genes involved in cytoplasm and cytoskeleton organisation allow for cellular stability, motility, and division, which are critical for maintaining both cellular health and broader functions required for organism functioning. These findings highlight the importance of core cellular mechanisms in driving the ageing process of the skin.

## Limitations

A significant challenge in this study is the heterogeneity across the included studies, stemming from differences in study design, population demographics, phenotype definitions, and the specific SNPs assessed within the same locus. Despite exhaustive efforts to include a wide range of studies, the majority still focused on European and East Asian populations, limiting the generalisability of the findings to other ethnic groups. Additionally, most studies relied on visual assessments of skin phenotypes, which, while cost-effective, are inherently subjective and susceptible to personal and cultural biases.

## Conclusion

This review has compiled data on genetic factors that have been found to be associated with a variety of skin ageing phenotypes from the 48 GWAS and candidate gene studies. Our findings showed that skin ageing genes tend to be involved in fundamental biological processes such as development, cytoskeleton and cytoplasm organisation, and pigmentation. The observed associations suggest that the pathogenesis of skin ageing involves core cellular mechanisms that are essential for the maintenance and function of cells, reinforcing the complexity of the ageing process. The pattern of certain loci tending to be associated with a certain category of phenotypes also reveals which biological pathways and mechanisms can be related to the pathogenesis of a certain category of skin ageing. However, further research is needed to better understand the specific pathways and mechanisms through which these genetic factors influence skin ageing.

## Supplementary Information

Additional file 1. PRISMA 2020 for Abstracts Checklist

Additional file 2. PRISMA 2020 Checklist

Additional file 3. Full set of results from functional enrichment with exclusion

Additional file 4. Full list of studies and study characteristics that were included from the literature search

Additional file 5. List of loci found to be associated with skin ageing in multiple studies

Additional file 6. Forest plot of *AFG3L1P.*

Additional file 7. Forest plot of intergenic region between *ASIP* and *ENSG00000287853.*

Additional file 8. Forest plot of *CPNE7.*

Additional file 9. Forest plot of *CPNE7* and *DPEP1.*

Additional file 10. Forest plot of *DEF8.*

Additional file 11. Forest plot of intergenic region between *DEF8* and *TUBB3.*

Additional file 12. Forest plot of *ENSG00000259006.*

Additional file 13. Forest plot of the intergenic region between *ENSG00000286364.*

Additional file 14. Forest plot of *IRF4.*

Additional file 15. Forest plot of *SPG7.*

Additional file 16. Forest plot of intergenic region between *TYR.*

Additional file 17. Forest plot of *BCAR3.*

Additional file 18. Forest plot of *ENSG00000233635.*

Additional file 19. Forest plot of *ENSG00000242593.*

Additional file 20. Forest plot of *MYO16.*

Additional file 21. Forest plot of *PRDM16.*

Additional file 22. Forest plot of *RORA.*

Additional file 23. Forest plot of *SPTLC1.*

Additional file 24. Forest plot of intergenic region between *BNC2* and *ENSG00000237153.*

Additional file 25. Forest plot of *CLPTM1L.*

Additional file 26. Forest plot of intergenic region between *ENSG00000271399* and *ENSG00000271475.*

Additional file 27. Forest plot of *LPP.*

Additional file 28. Forest plot of *BPIFA3.*

Additional file 29. Forest plot of intergenic region between *DYSF* and *RPS20P10.*

Additional file 30. Forest plot of intergenic region between *ENSG00000241168* and *ENSG00000243044.*

Additional file 31. Forest plot of intergenic region between *ENSG00000286364* and *IRF4.*

Additional file 32. Forest plot of *ENSG00000286803.*

Additional file 33. Forest plot of *NCOA6.*

Additional file 34. Forest plot of *SPON1.*

Additional file 35. Forest plot of *SV2B.*

Additional file 36. Forest plot of *ANKRD11.*

Additional file 37. Forest plot of *CDK10.*

Additional file 38. Forest plot of *DBNDD1.*

Additional file 39. Forest plot of *GAS8.*

Additional file 40. Forest plot of *FAR2.*

Additional file 41. Forest plot of *TANC2.*

## Data Availability

All data generated or analysed during this study are included in this published article and its supplementary information files. Further inquiries about the datasets used and/or analysed during the current study can be directed to the corresponding author (F. T. C.) on reasonable request. The 48 publications selected for the review were identified through the primary search and remained relevant to the aims of this review after a subsequent screening process. The results of the functional enrichment are reported in Additional File 3. The publications are reported in Additional File 4. The characteristics of all 95 loci that were used for meta-analysis are reported in Additional File 5. All 30 forest plots that had overall significant association were included in Additional files 6, 7, 8, 9, 10, 11, 12, 13, 14, 15, 16, 17, 18, 19, 20, 21, 22, 23, 24, 25, 26, 27, 28, 29, 30, 31, 32, 33, 34, 35, 36, 37, 38, 39, 40, and 41. A review protocol was not prepared prior to the writing of this manuscript, but the review methodology follows what was described in the ‘Methods’ section.

## Abbreviations

*AFG3L1P*: AFG3-like matrix AAA peptidase subunit 1
*ANKRD11*: Ankyrin repeat domain 11
*ASIP*: Agouti-signalling protein
*BCAR3*: Breast cancer anti-oestrogen resistance 3
*BNC2*: Basonuclin zinc-finger protein 2
*CDC42BPA*: CDC42-binding protein kinase alpha
*CDK10*: Cyclin-dependent kinase 10
CI: Confidence interval
*CLPTM1L*: CLPTM1L like
*CPNE7*: Copine 7
*DBNDD1*: Dysbindin domain containing 1
*DEF8*: Differentially expressed in FDCP 8 homolog
ENSG: Ensembl Stable ID
*FANCA*: FA complementation group A
*FAR2*: Fatty acyl-CoA reductase 2
*GAS8*: Growth arrest specific 8
gDNA: Genomic DNA
GWAS: Genome-wide association study
*HERC2*: HECT and RLD domain containing E3 ubiquitin protein ligase 2
*IRF4*: Interferon regulatory factor 4
*LPP*: LIM domain containing preferred translocation partner in lipoma
*MC1R*: Melanocortin 1 receptor
*MFSD12*: Major facilitator superfamily domain containing 12
*MYO16*: Myosin XVI
*OCA2*: OCA2 melanosomal transmembrane protein
OR: Odds Ratio
*PRDM7*: PR/SET domain 7
*PPARGC1B*: PPARG coactivator 1 beta
*PRDM16*: PR/SET domain 16
PRISMA: Preferred Reporting Item for Systematic review and Meta-Analyses
ROS: Reactive oxygen species
*RORA*: RAR-related orphan receptor A
*SMYD3*: SET and MYND domain-containing 3
SNP: Single-nucleotide polymorphism
*SPG7*: SPG7 matrix AAA peptidase subunit
*SPIRE2*: Spire type actin nucleation factor 2
*SPTLC1*: Serine palmitoyltransferase long chain base subunit 1
*TANC2*: Tetratricopeptide repeat, ankyrin repeat, and coiled-coil containing 2
*TAU*: Tubulin-associated unit
*TUBB3*: Tubulin beta 3 class III
*TYR*: Tyrosinase
WAVE: WASp-family verprolin homologous protein
