# Supplementary figures and images for: Genetic determinants of skin ageing: a systematic review and meta-analysis of genome-wide association studies and candidate genes
Source: J Physiol Anthropol. 2025 Feb 8;44:4. doi: 10.1186/s40101-025-00384-9 (PMC11806588; doi:10.1186/s40101-025-00384-9)

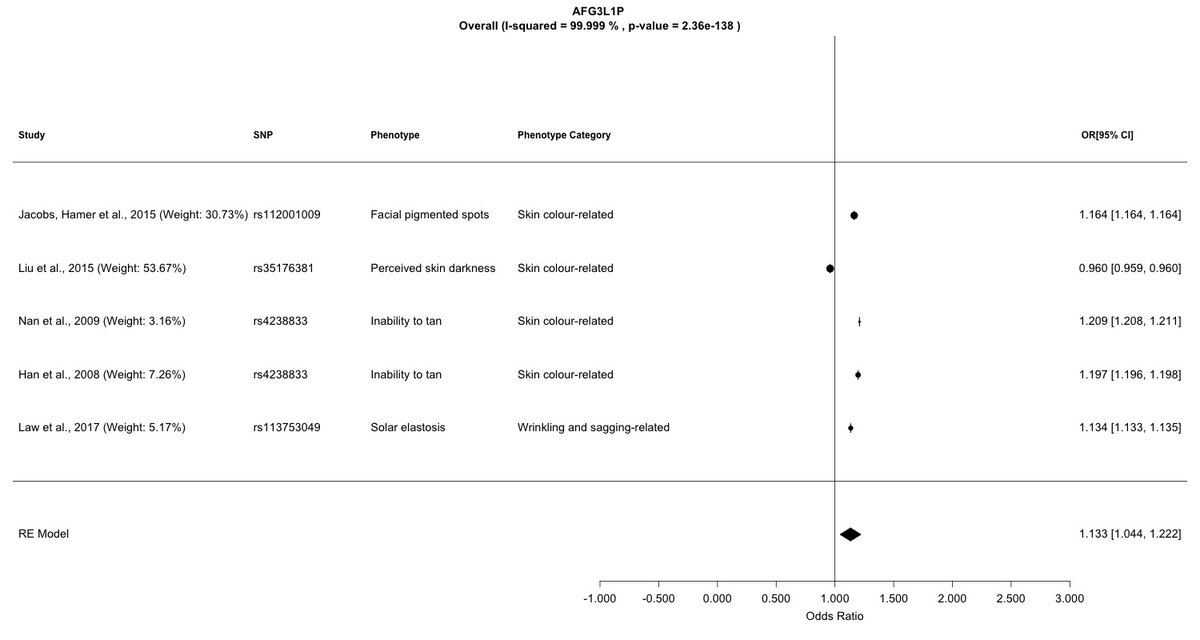

Supplement: Supplementary file 6 — Additional file 6. Forest plot of AFG3L1P. [file 40101_2025_384_MOESM6_ESM.png]

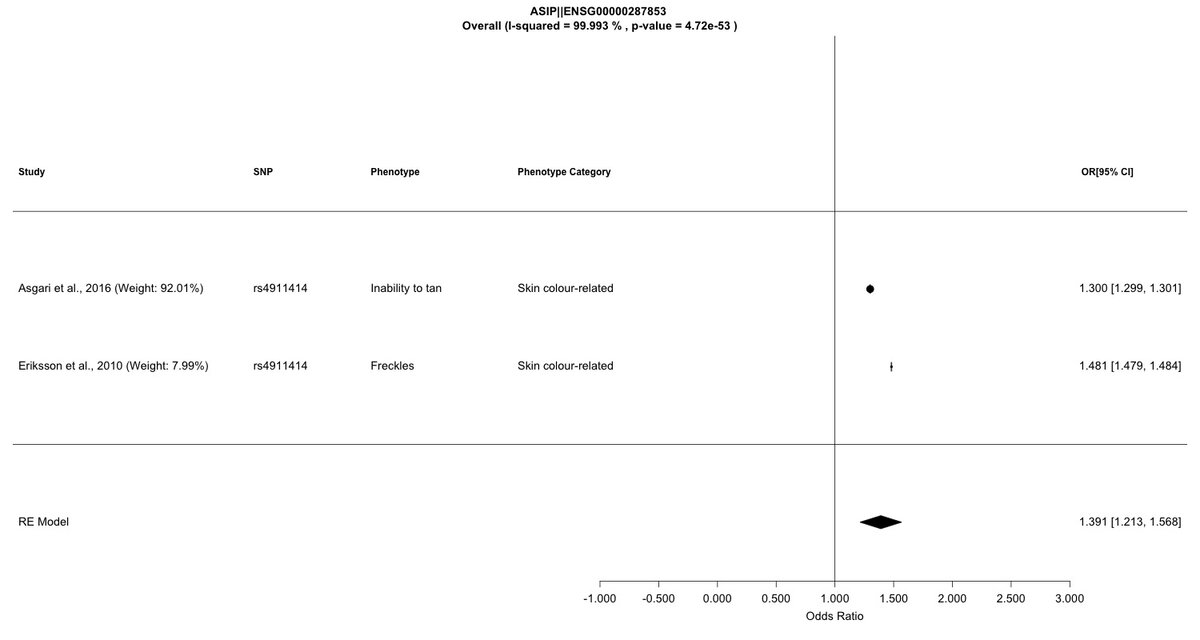

Supplement: Supplementary file 7 — Additional file 7. Forest plot of intergenic region between ASIP and ENSG00000287853. [file 40101_2025_384_MOESM7_ESM.png]

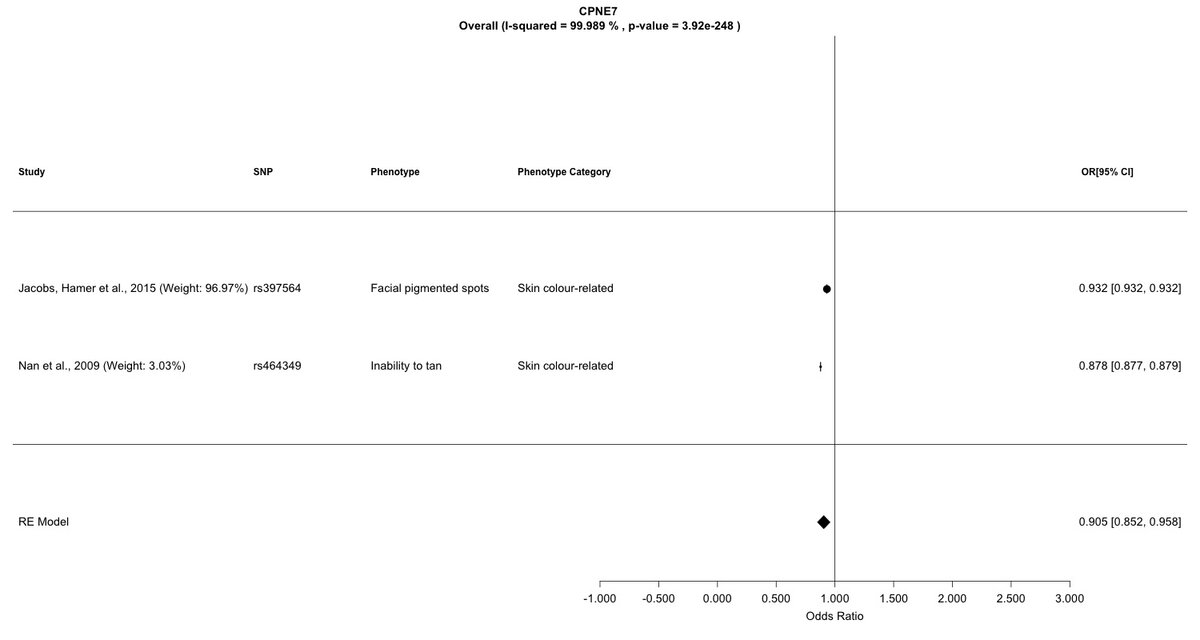

Supplement: Supplementary file 8 — Additional file 8. Forest plot of CPNE7. [file 40101_2025_384_MOESM8_ESM.png]

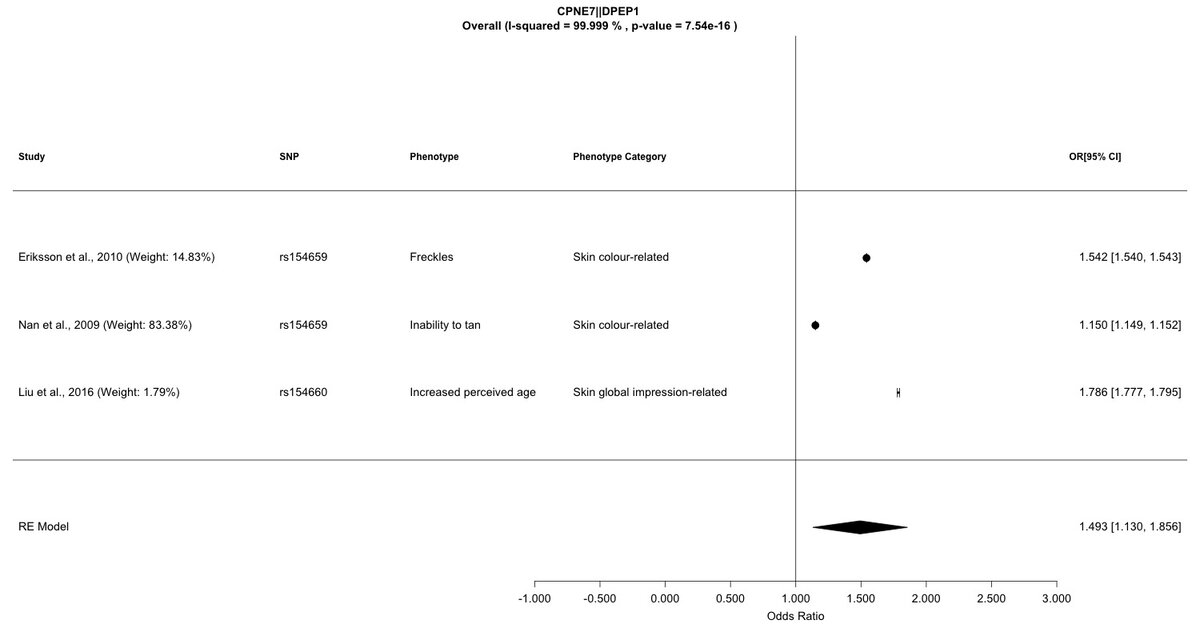

Supplement: Supplementary file 9 — Additional file 9. Forest plot of CPNE7 and DPEP1. [file 40101_2025_384_MOESM9_ESM.png]

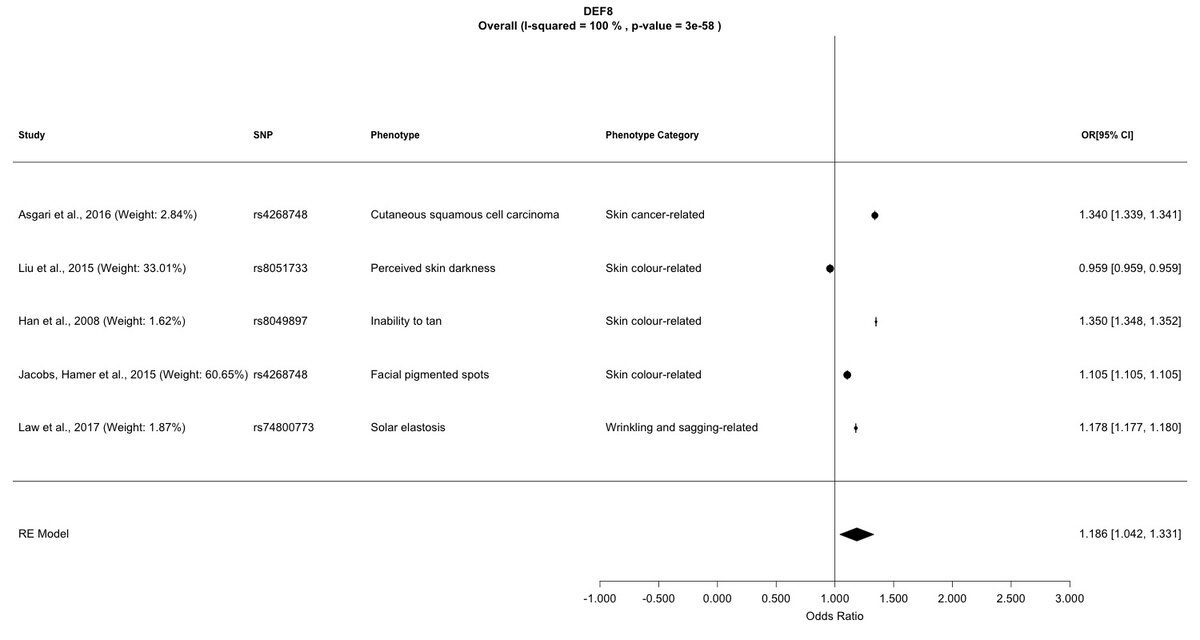

Supplement: Supplementary file 10 — Additional file 10. Forest plot of DEF8. [file 40101_2025_384_MOESM10_ESM.png]

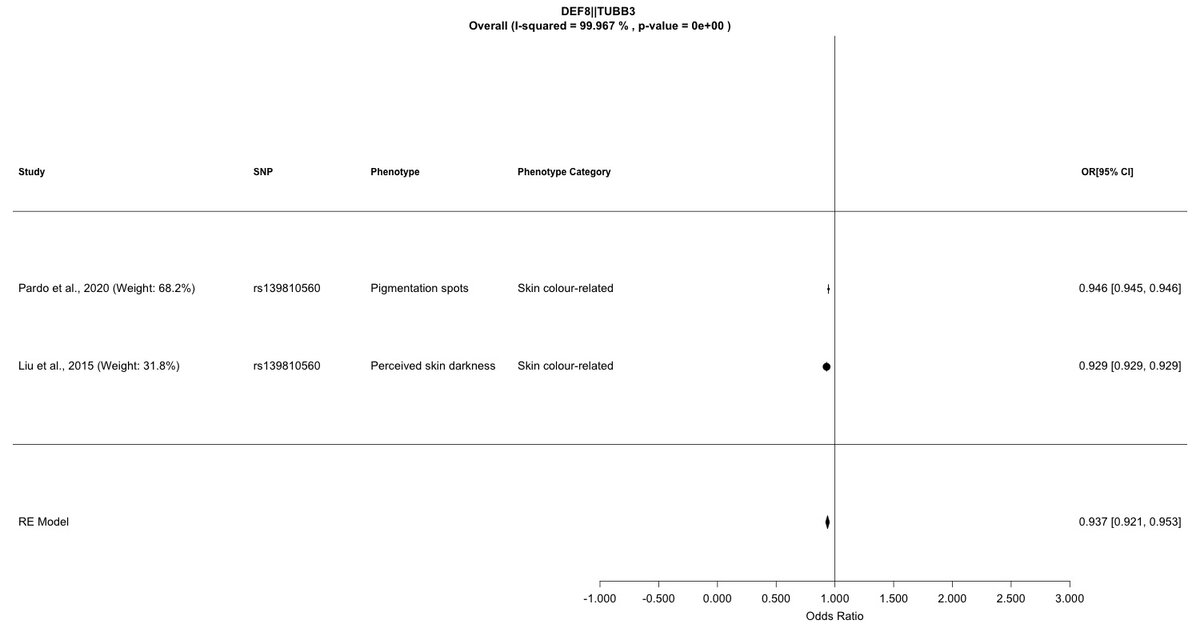

Supplement: Supplementary file 11 — Additional file 11. Forest plot of intergenic region between DEF8 and TUBB3. [file 40101_2025_384_MOESM11_ESM.png]

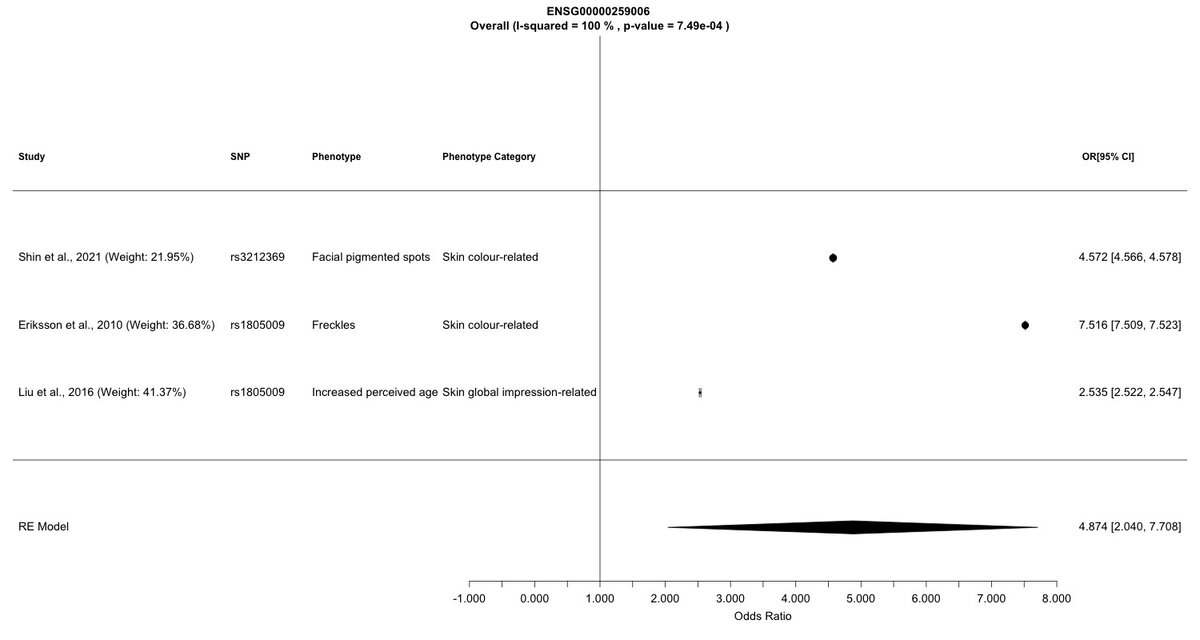

Supplement: Supplementary file 12 — Additional file 12. Forest plot of ENSG00000259006. [file 40101_2025_384_MOESM12_ESM.png]

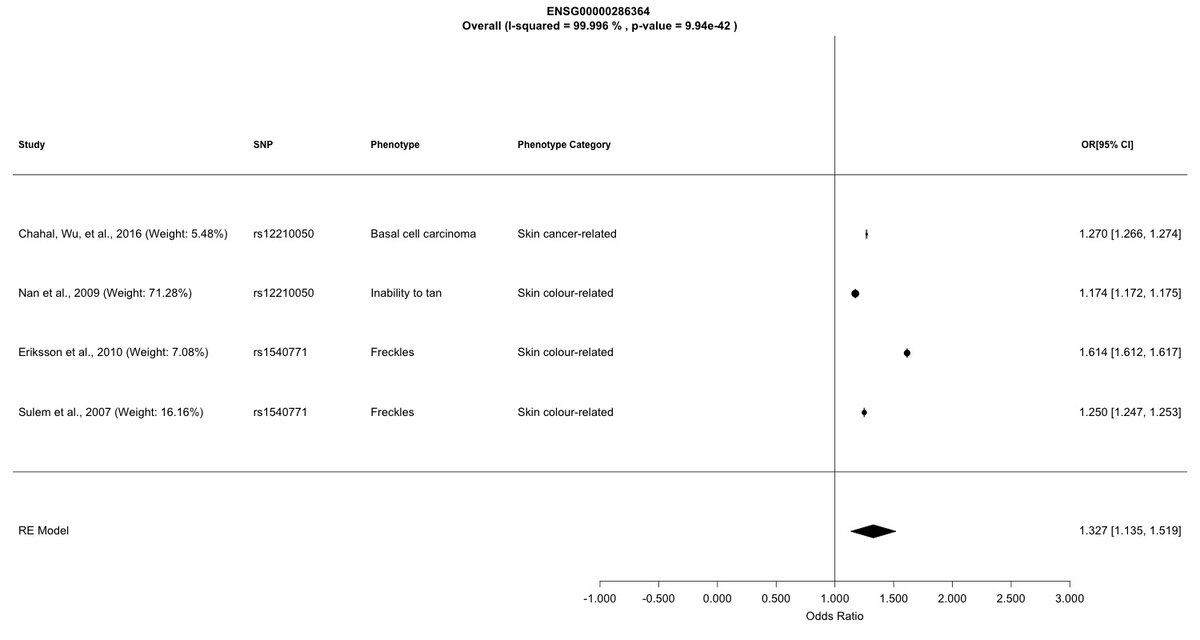

Supplement: Supplementary file 13 — Additional file 13. Forest plot of the intergenic region between ENSG00000286364. [file 40101_2025_384_MOESM13_ESM.png]

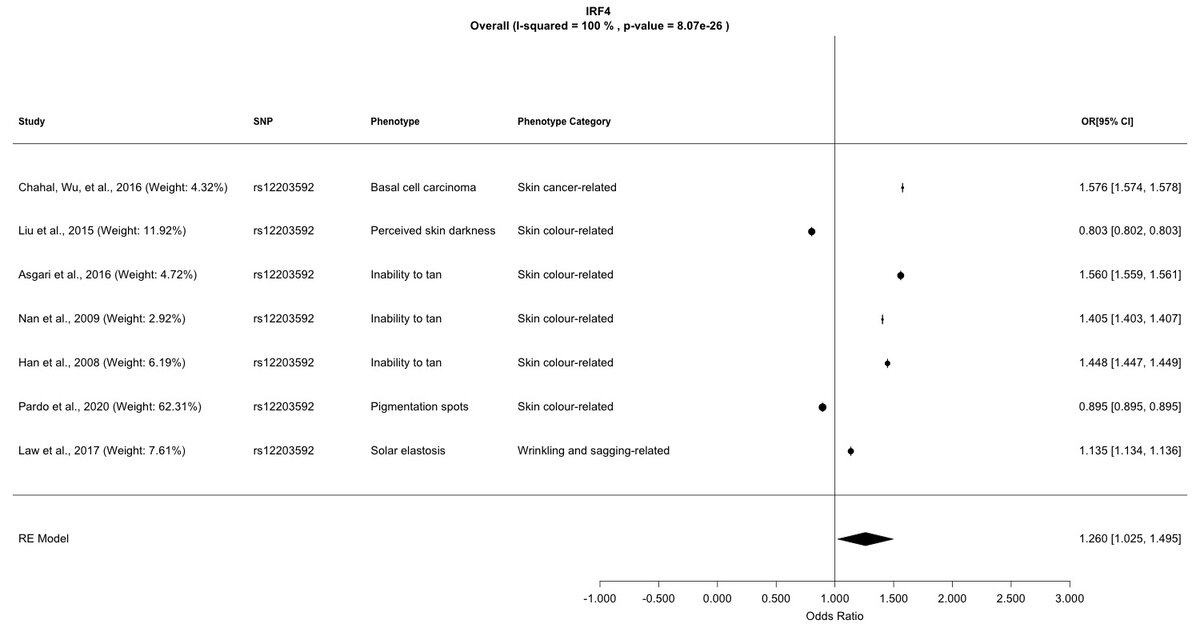

Supplement: Supplementary file 14 — Additional file 14. Forest plot of IRF4. [file 40101_2025_384_MOESM14_ESM.png]

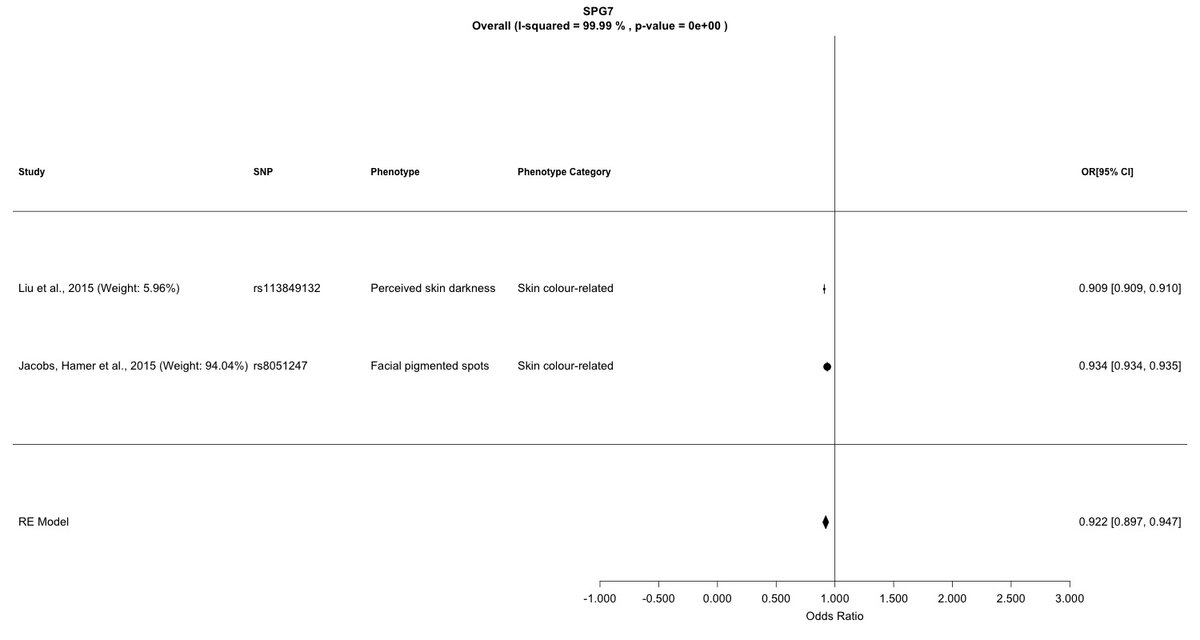

Supplement: Supplementary file 15 — Additional file 15. Forest plot of SPG7. [file 40101_2025_384_MOESM15_ESM.png]

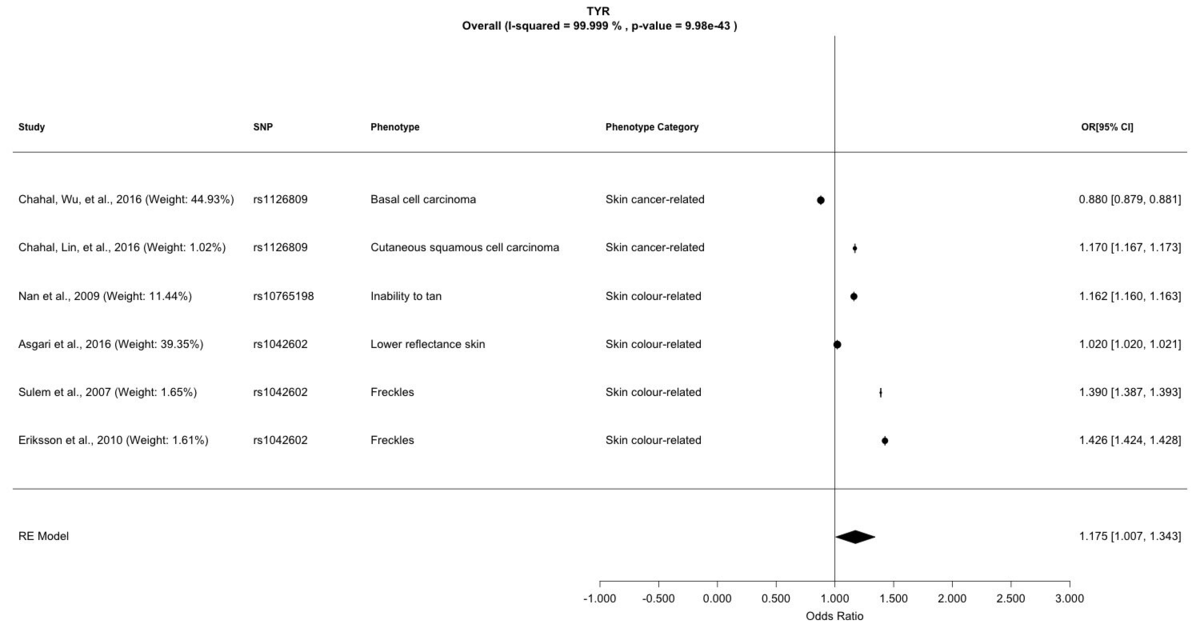

Supplement: Supplementary file 16 — Additional file 16. Forest plot of intergenic region between TYR. [file 40101_2025_384_MOESM16_ESM.png]

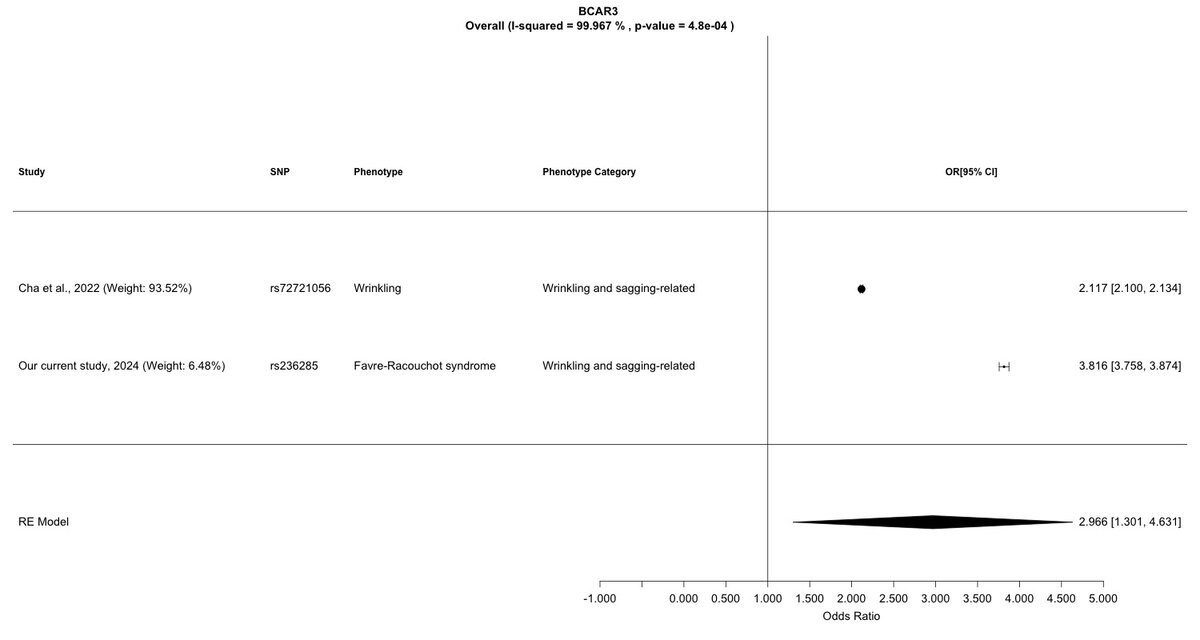

Supplement: Supplementary file 17 — Additional file 17. Forest plot of BCAR3. [file 40101_2025_384_MOESM17_ESM.png]

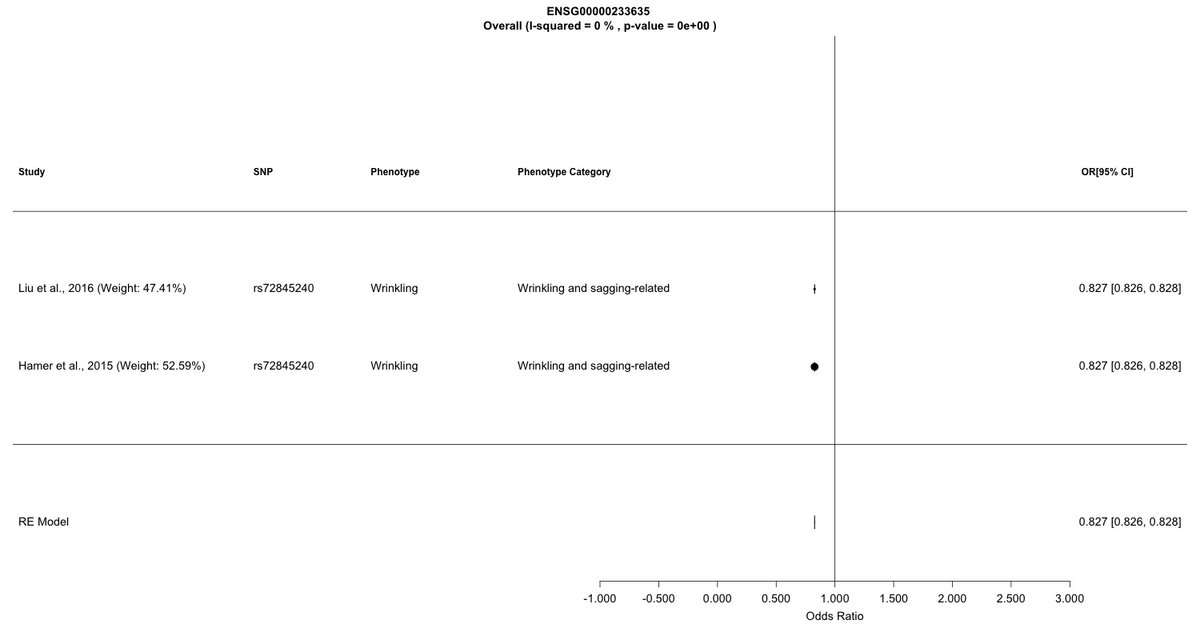

Supplement: Supplementary file 18 — Additional file 18. Forest plot of ENSG00000233635. [file 40101_2025_384_MOESM18_ESM.png]

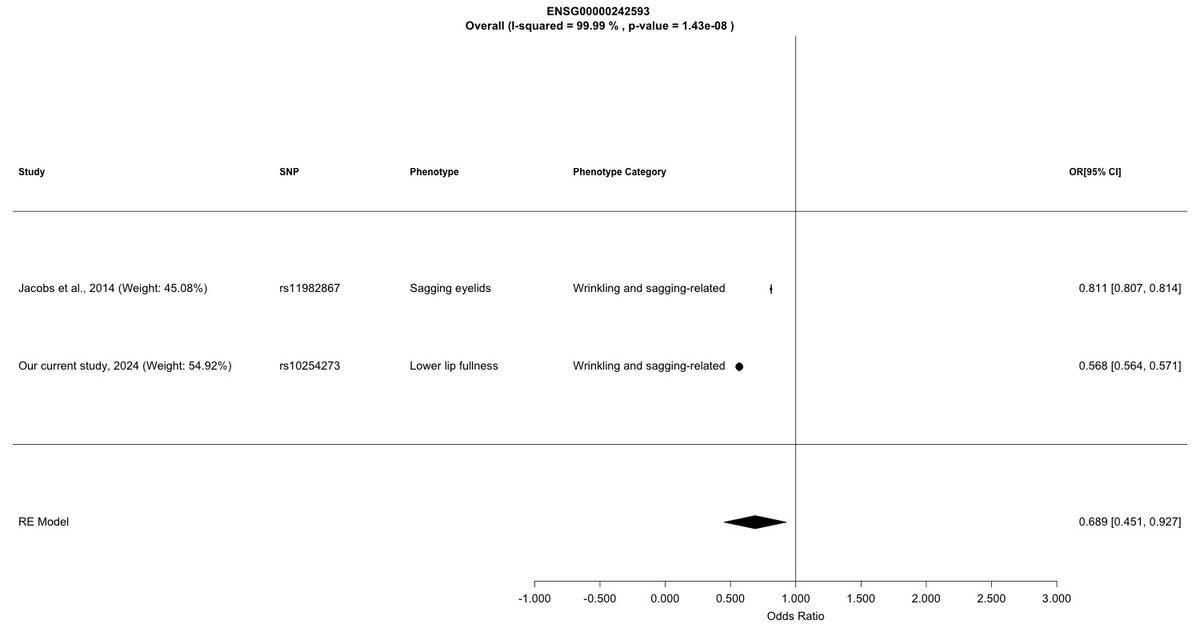

Supplement: Supplementary file 19 — Additional file 19. Forest plot of ENSG00000242593. [file 40101_2025_384_MOESM19_ESM.png]

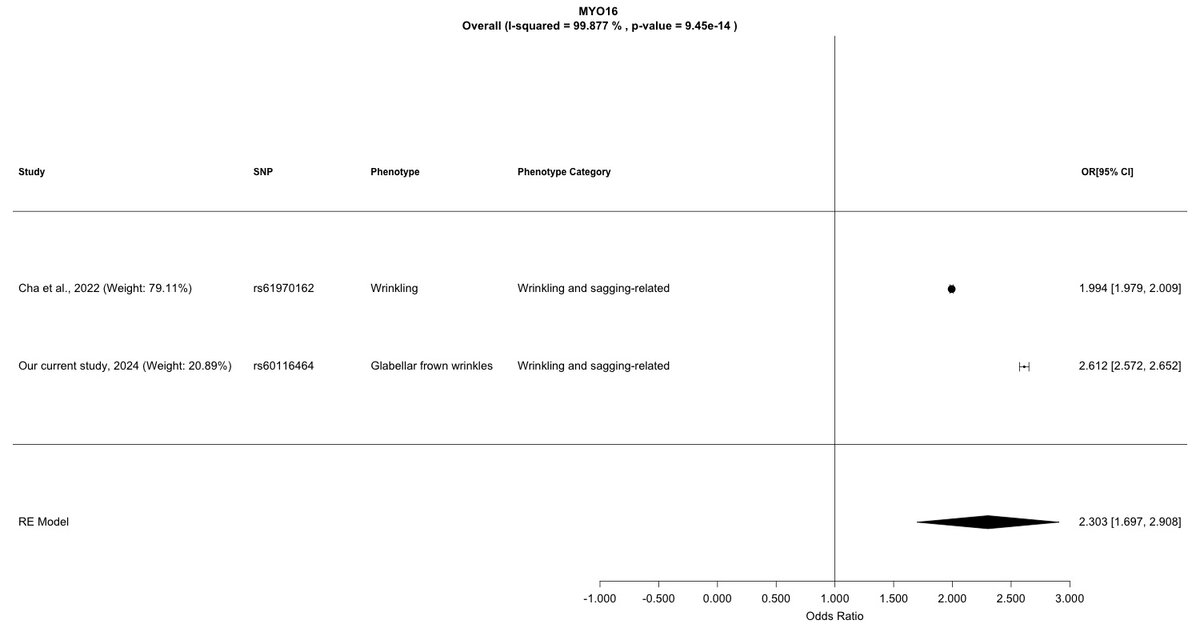

Supplement: Supplementary file 20 — Additional file 20. Forest plot of MYO16. [file 40101_2025_384_MOESM20_ESM.png]

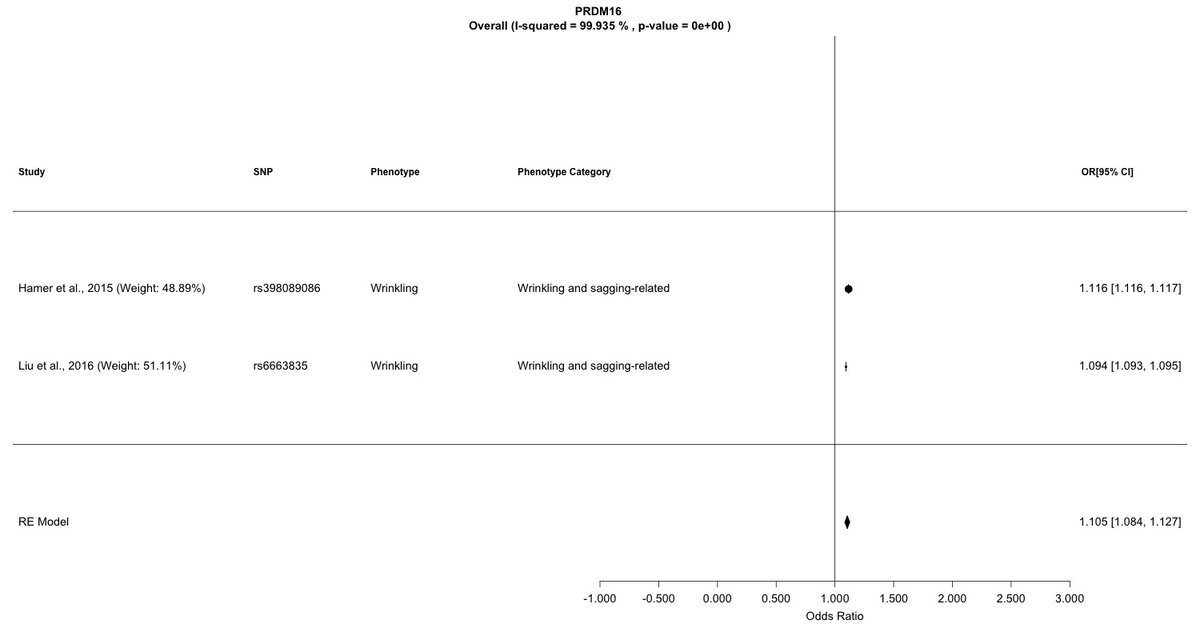

Supplement: Supplementary file 21 — Additional file 21. Forest plot of PRDM16. [file 40101_2025_384_MOESM21_ESM.png]

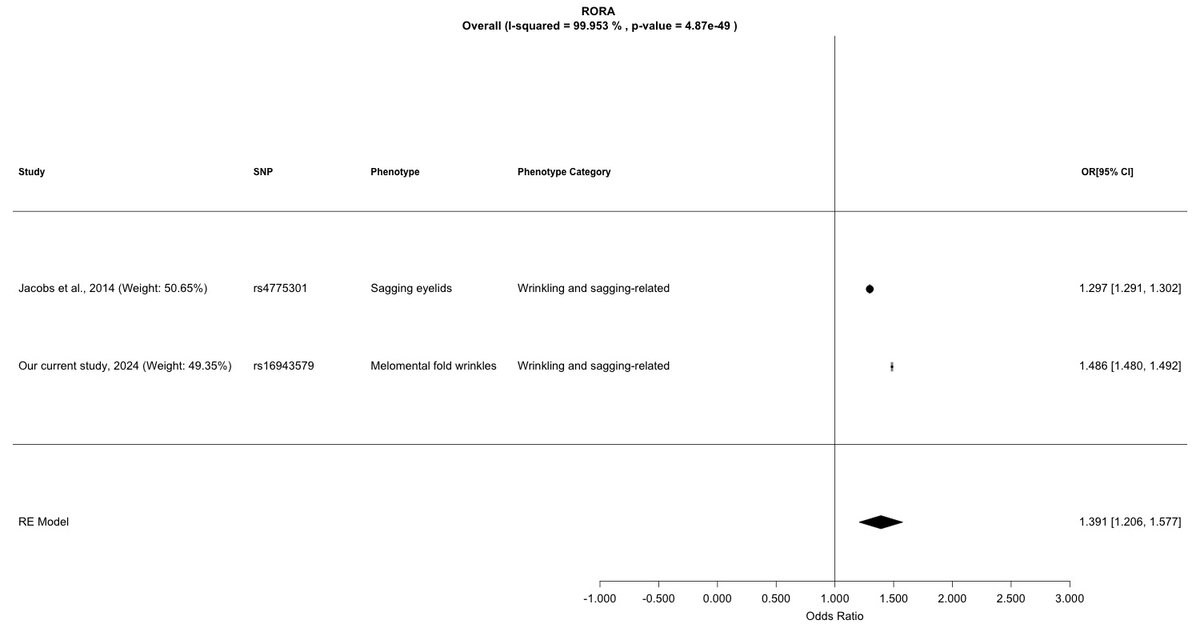

Supplement: Supplementary file 22 — Additional file 22. Forest plot of RORA. [file 40101_2025_384_MOESM22_ESM.png]

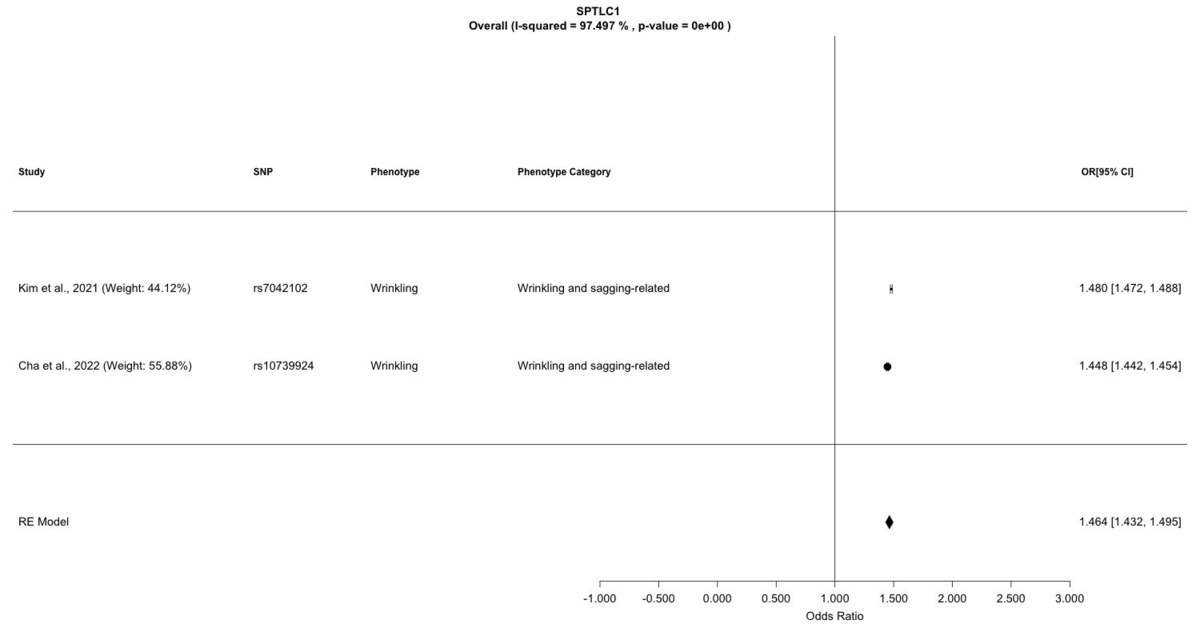

Supplement: Supplementary file 23 — Additional file 23. Forest plot of SPTLC1. [file 40101_2025_384_MOESM23_ESM.png]

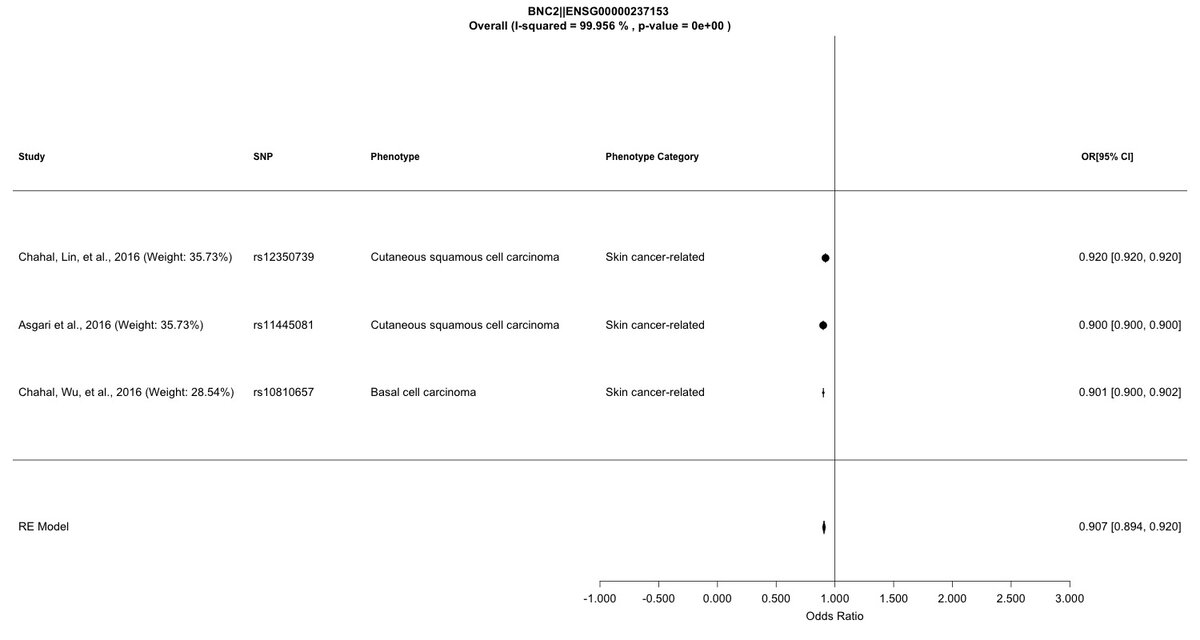

Supplement: Supplementary file 24 — Additional file 24. Forest plot of intergenic region between BNC2 and ENSG00000237153. [file 40101_2025_384_MOESM24_ESM.png]

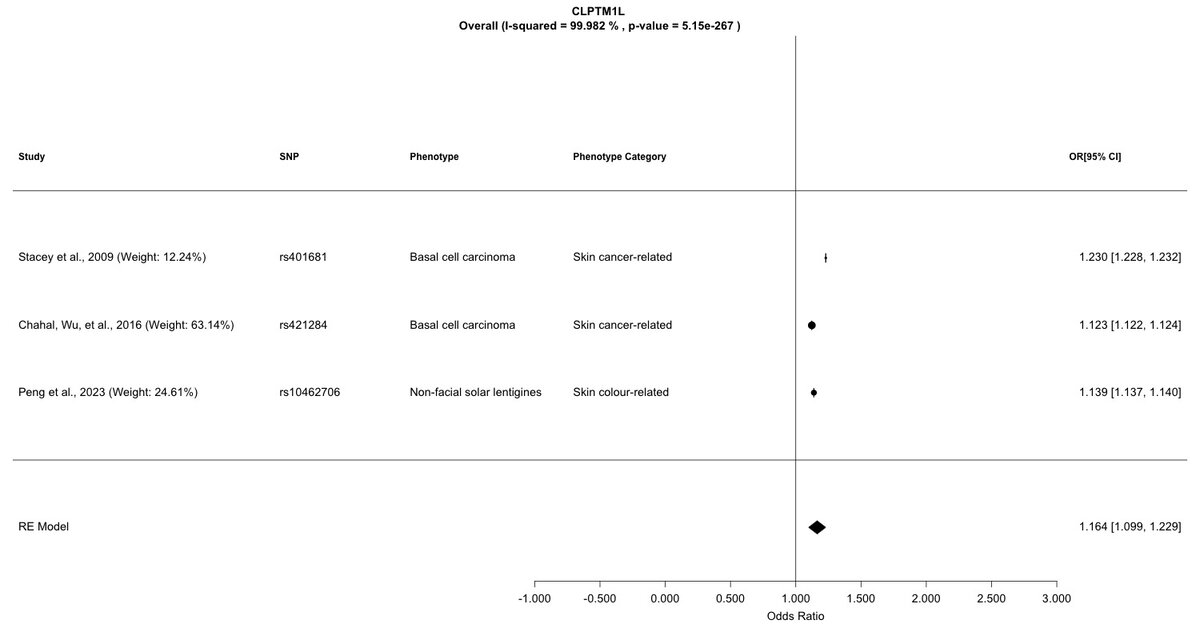

Supplement: Supplementary file 25 — Additional file 25. Forest plot of CLPTM1L. [file 40101_2025_384_MOESM25_ESM.png]

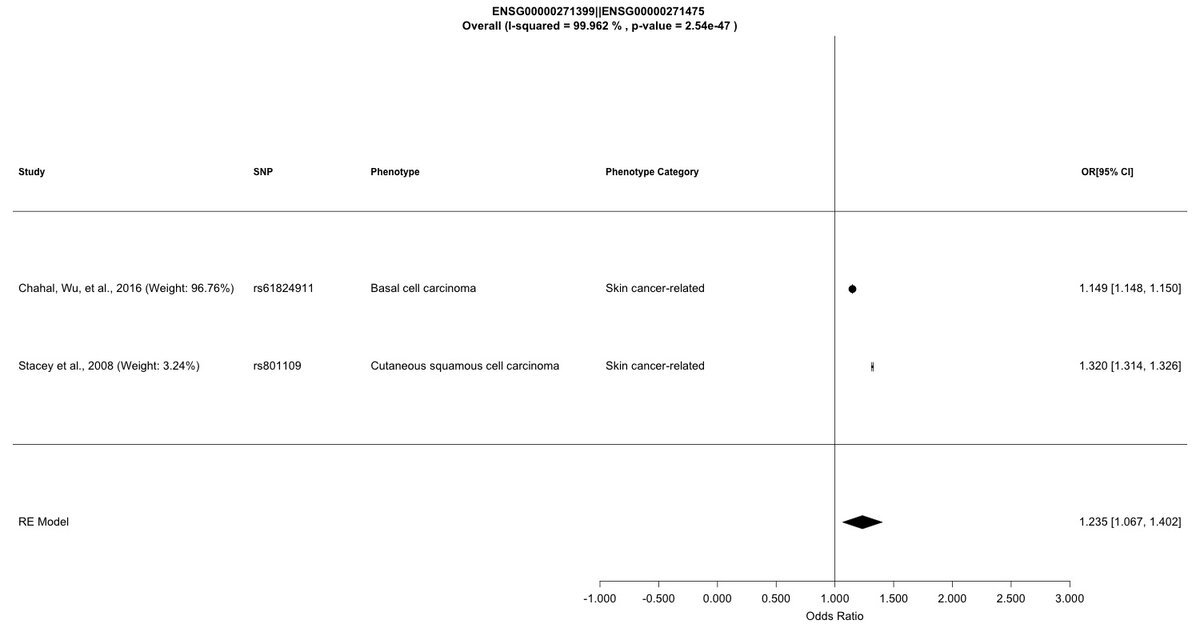

Supplement: Supplementary file 26 — Additional file 26. Forest plot of intergenic region between ENSG00000271399 and ENSG00000271475. [file 40101_2025_384_MOESM26_ESM.png]

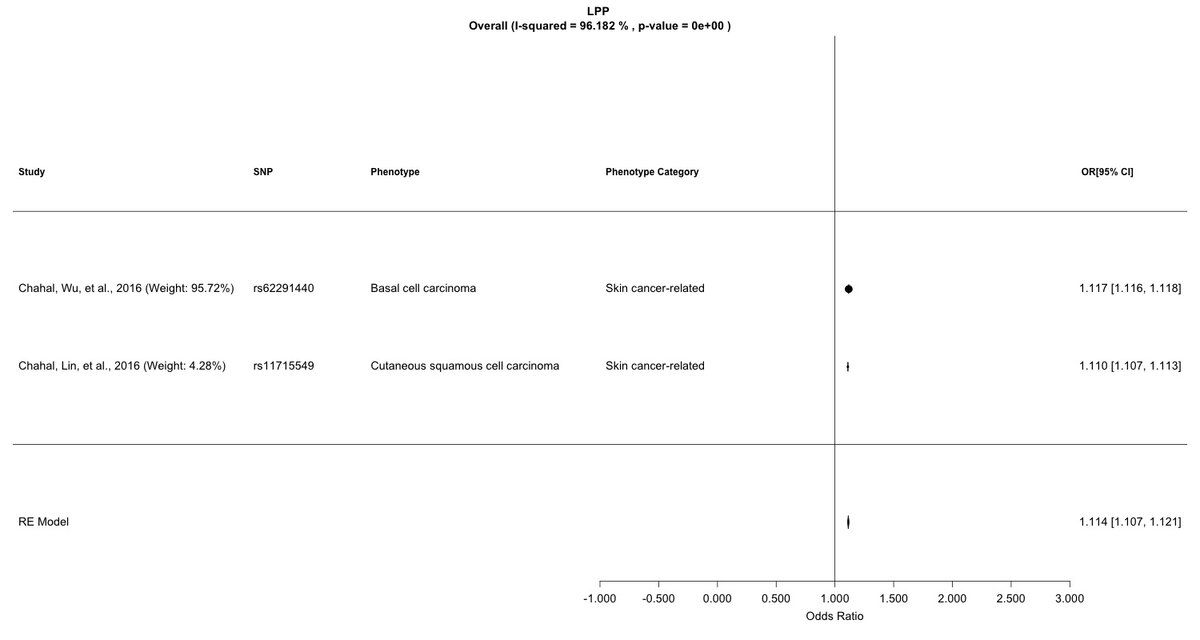

Supplement: Supplementary file 27 — Additional file 27. Forest plot of LPP. [file 40101_2025_384_MOESM27_ESM.png]

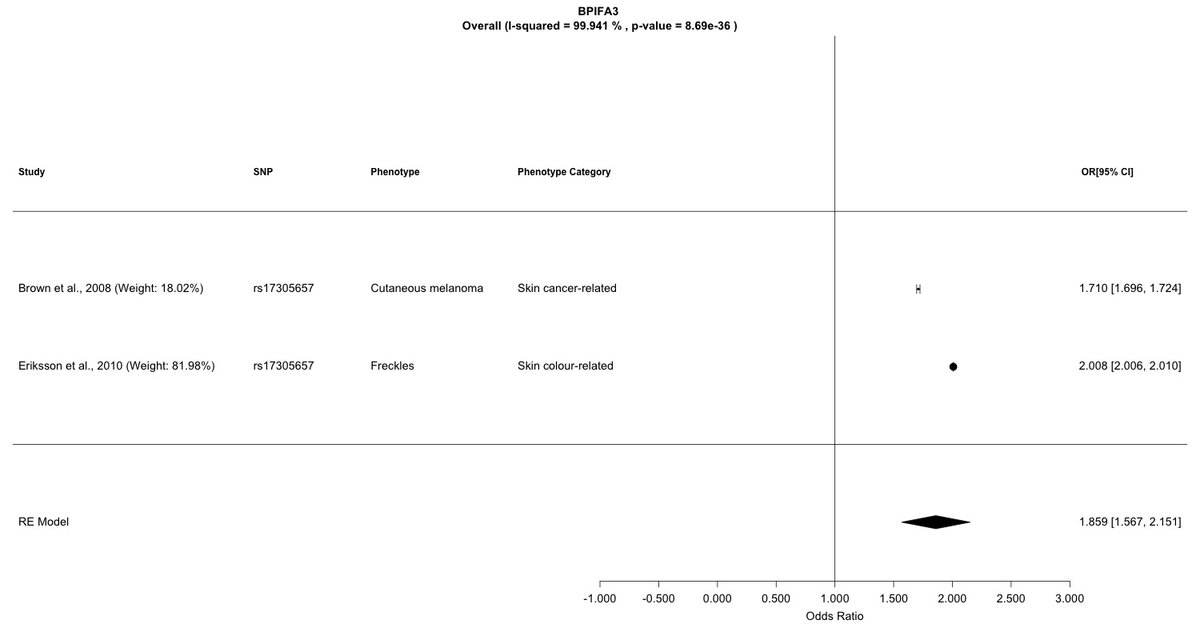

Supplement: Supplementary file 28 — Additional file 28. Forest plot of BPIFA3. [file 40101_2025_384_MOESM28_ESM.png]

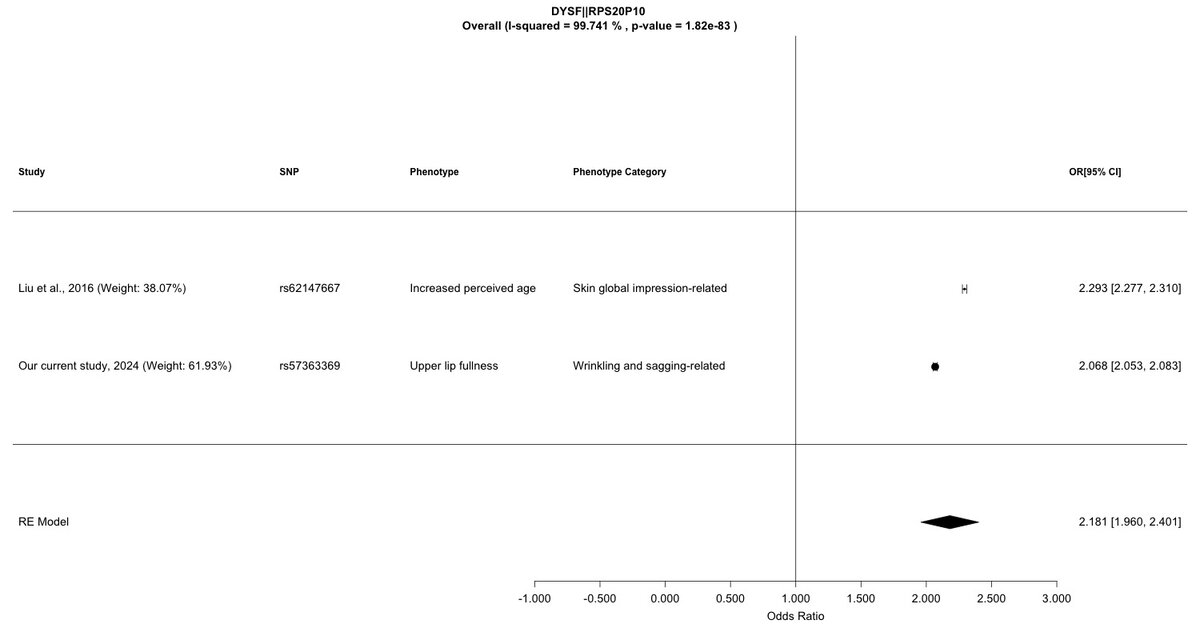

Supplement: Supplementary file 29 — Additional file 29. Forest plot of intergenic region between DYSF and RPS20P10. [file 40101_2025_384_MOESM29_ESM.png]

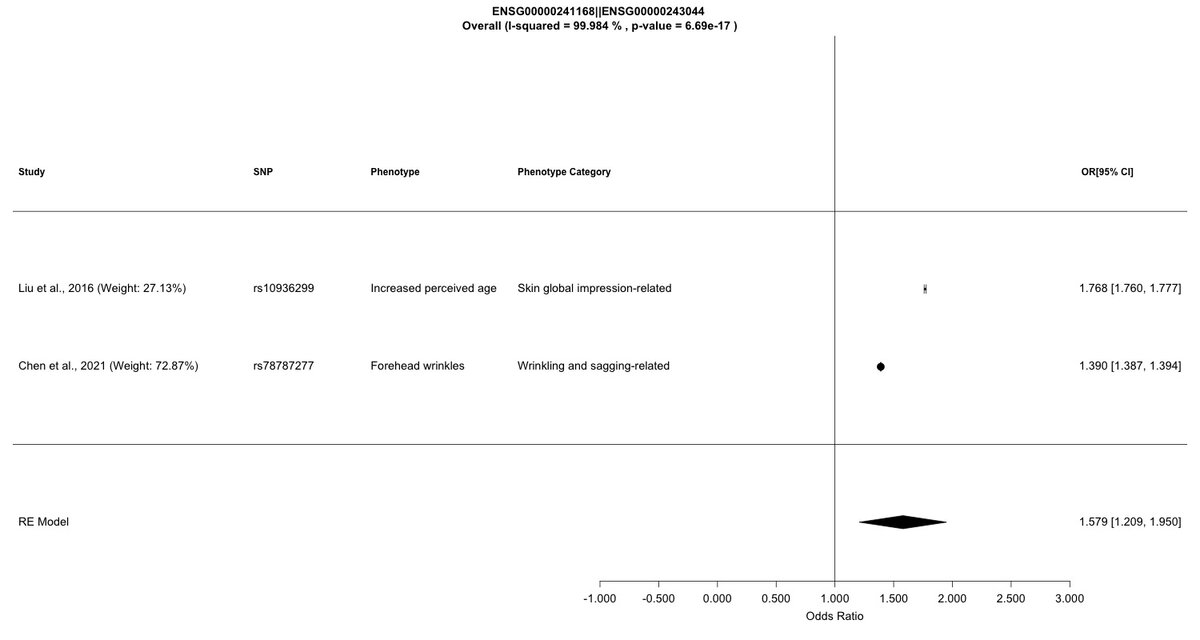

Supplement: Supplementary file 30 — Additional file 30. Forest plot of intergenic region between ENSG00000241168 and ENSG00000243044. [file 40101_2025_384_MOESM30_ESM.png]

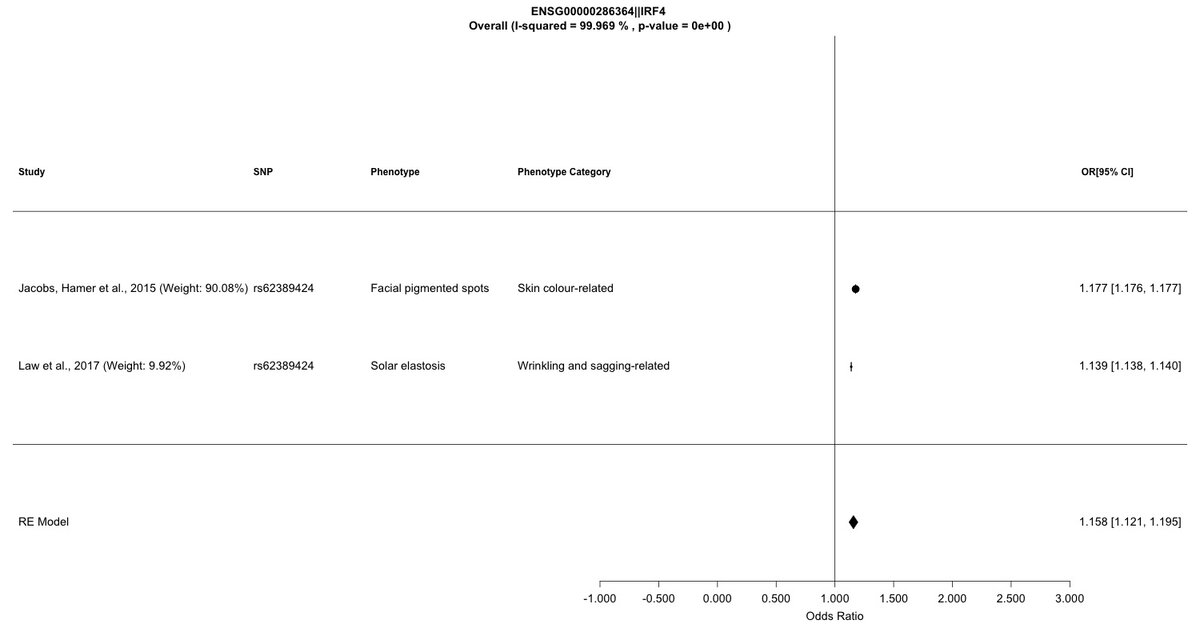

Supplement: Supplementary file 31 — Additional file 31. Forest plot of intergenic region between ENSG00000286364 and IRF4. [file 40101_2025_384_MOESM31_ESM.png]

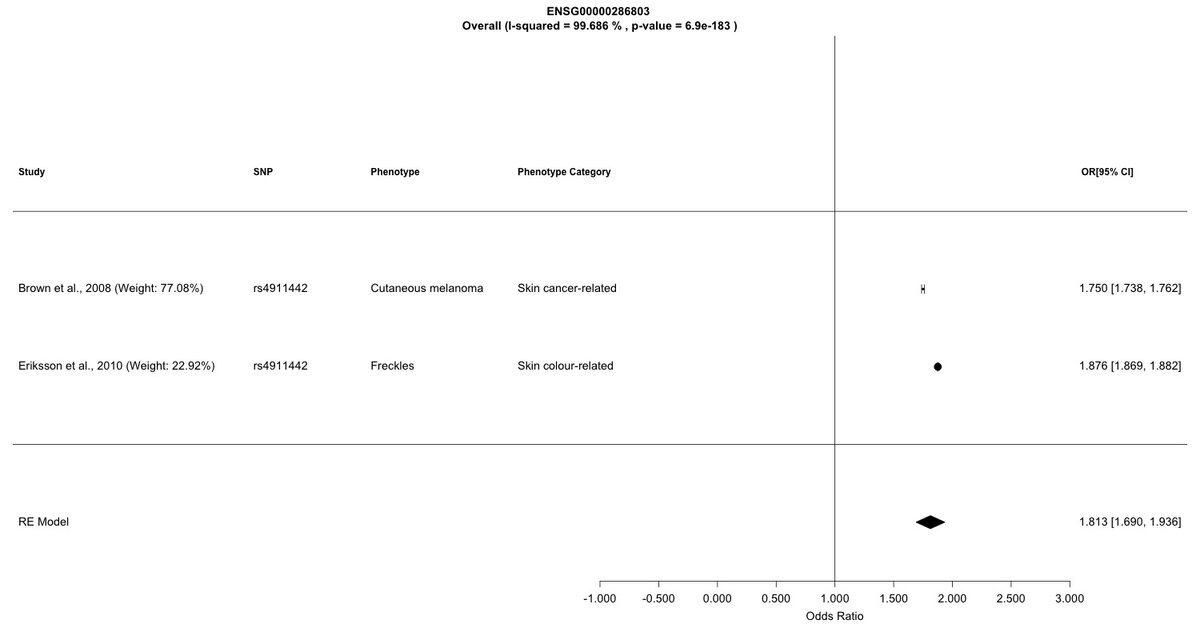

Supplement: Supplementary file 32 — Additional file 32. Forest plot of ENSG00000286803. [file 40101_2025_384_MOESM32_ESM.png]

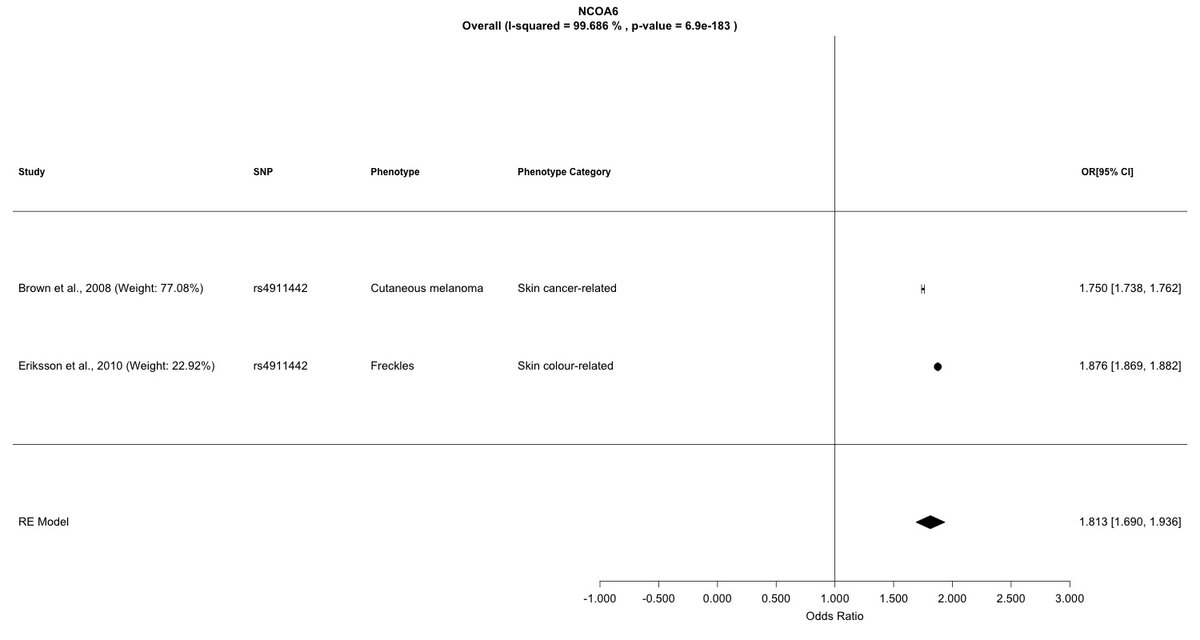

Supplement: Supplementary file 33 — Additional file 33. Forest plot of NCOA6. [file 40101_2025_384_MOESM33_ESM.png]

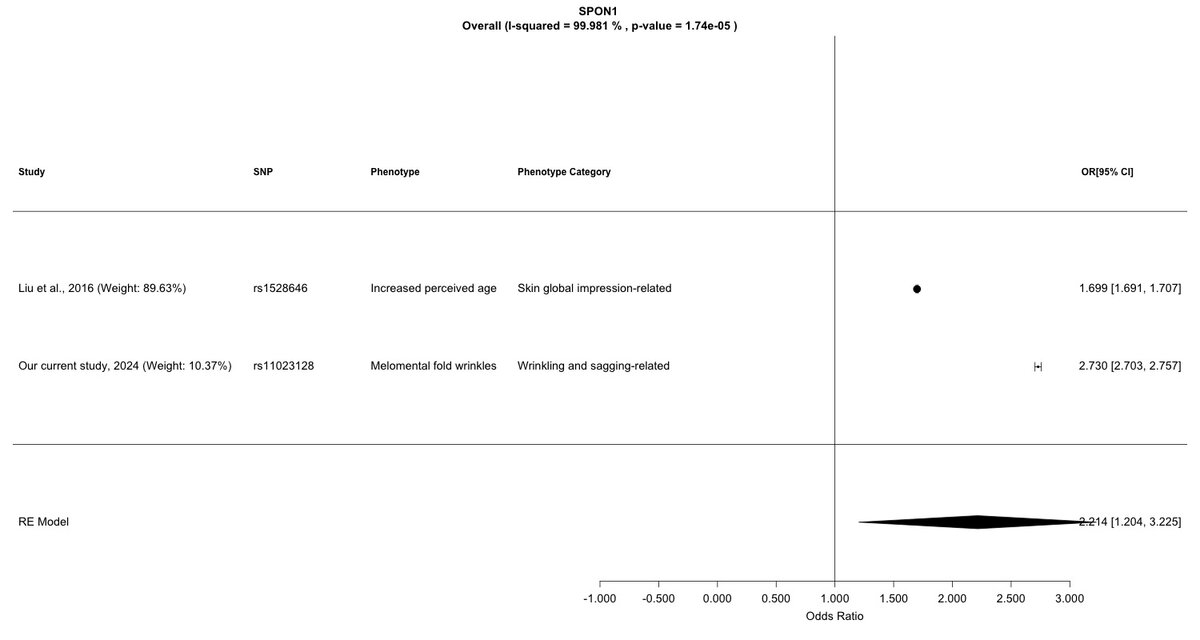

Supplement: Supplementary file 34 — Additional file 34. Forest plot of SPON1. [file 40101_2025_384_MOESM34_ESM.png]

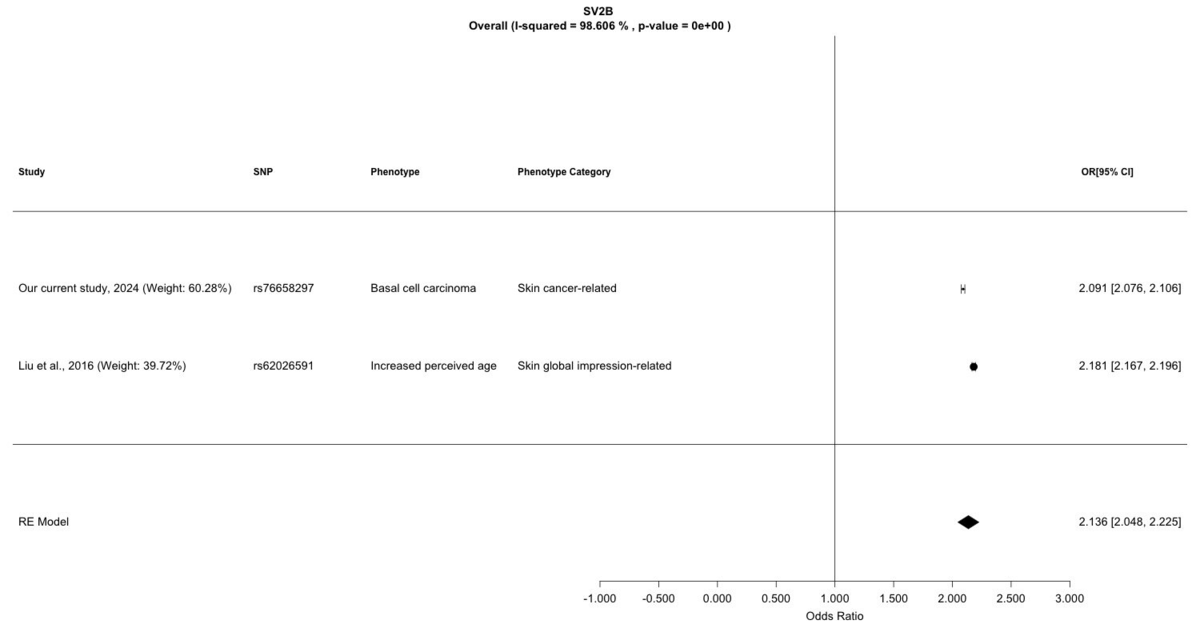

Supplement: Supplementary file 35 — Additional file 35. Forest plot of SV2B. [file 40101_2025_384_MOESM35_ESM.png]

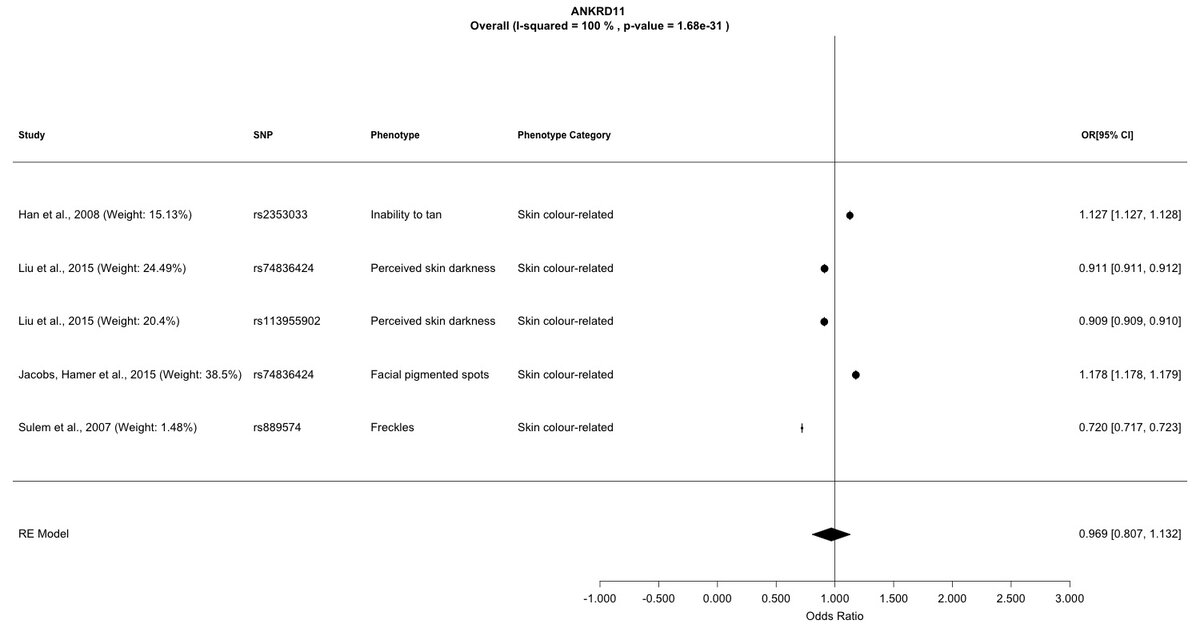

Supplement: Supplementary file 36 — Additional file 36. Forest plot of ANKRD11. [file 40101_2025_384_MOESM36_ESM.png]

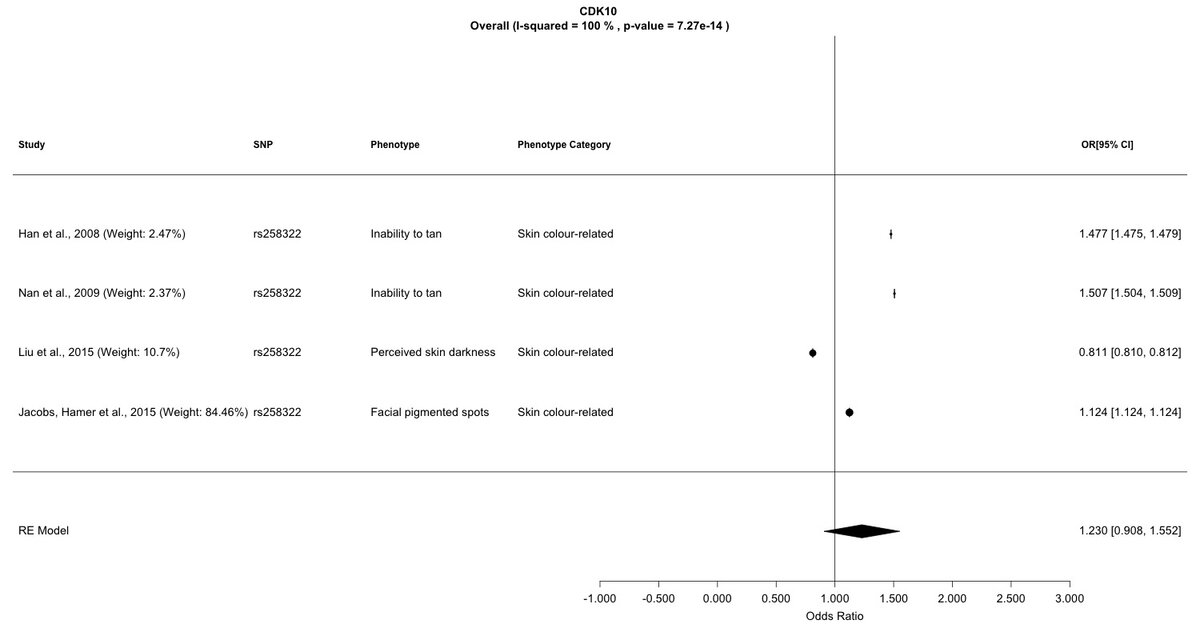

Supplement: Supplementary file 37 — Additional file 37. Forest plot of CDK10. [file 40101_2025_384_MOESM37_ESM.png]

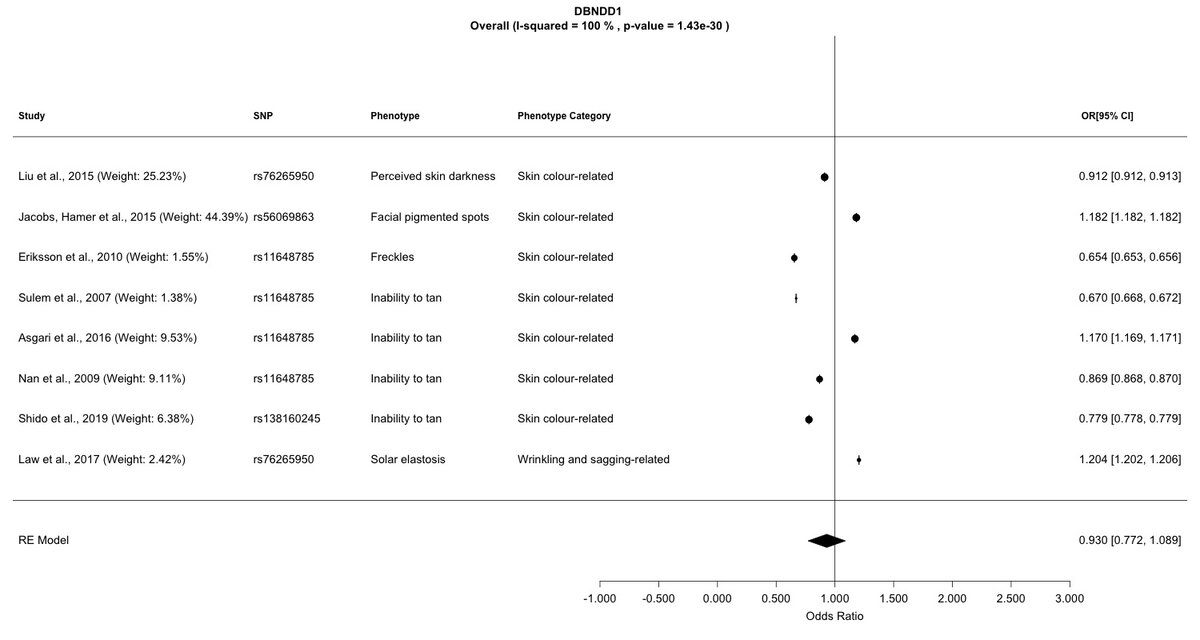

Supplement: Supplementary file 38 — Additional file 38. Forest plot of DBNDD1. [file 40101_2025_384_MOESM38_ESM.png]

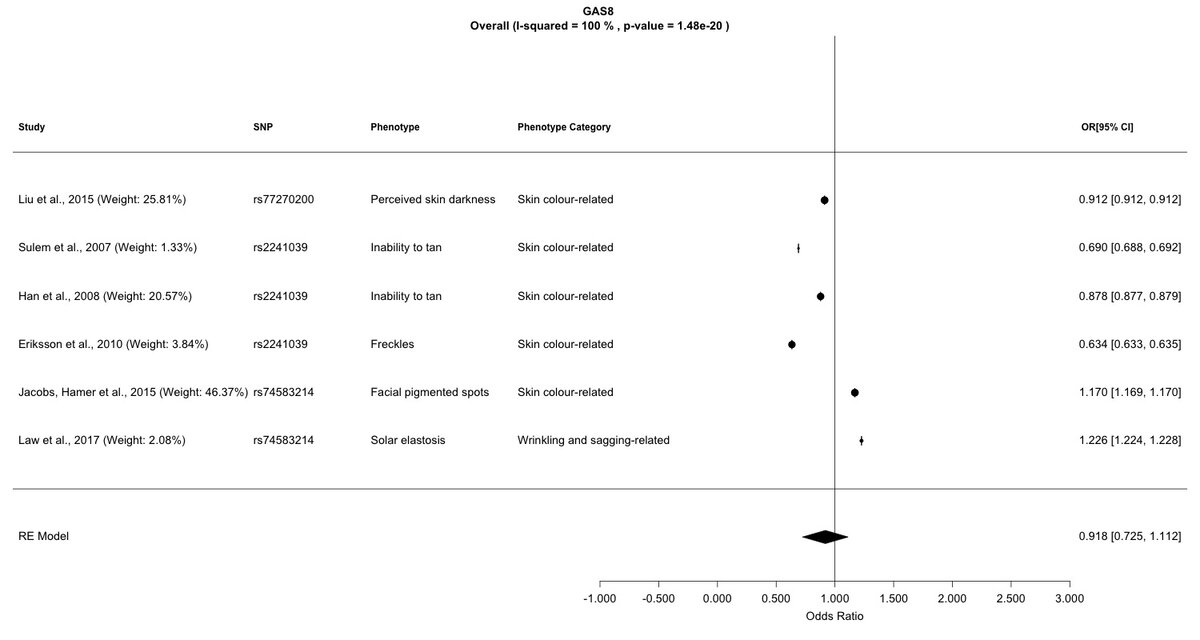

Supplement: Supplementary file 39 — Additional file 39. Forest plot of GAS8. [file 40101_2025_384_MOESM39_ESM.png]

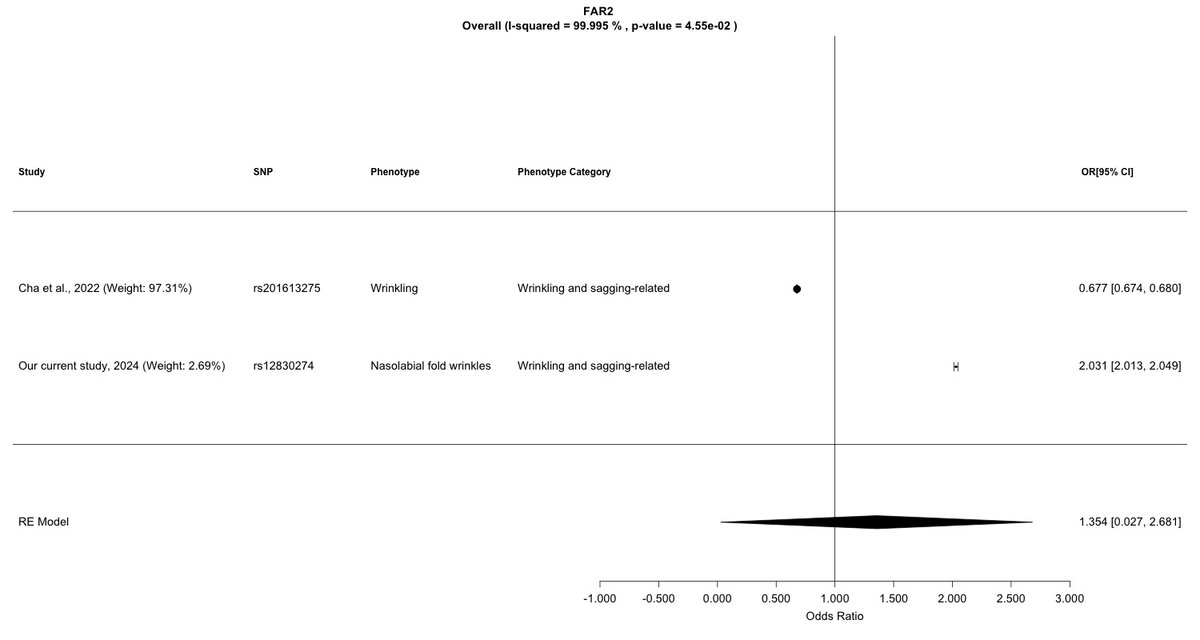

Supplement: Supplementary file 40 — Additional file 40. Forest plot of FAR2. [file 40101_2025_384_MOESM40_ESM.png]

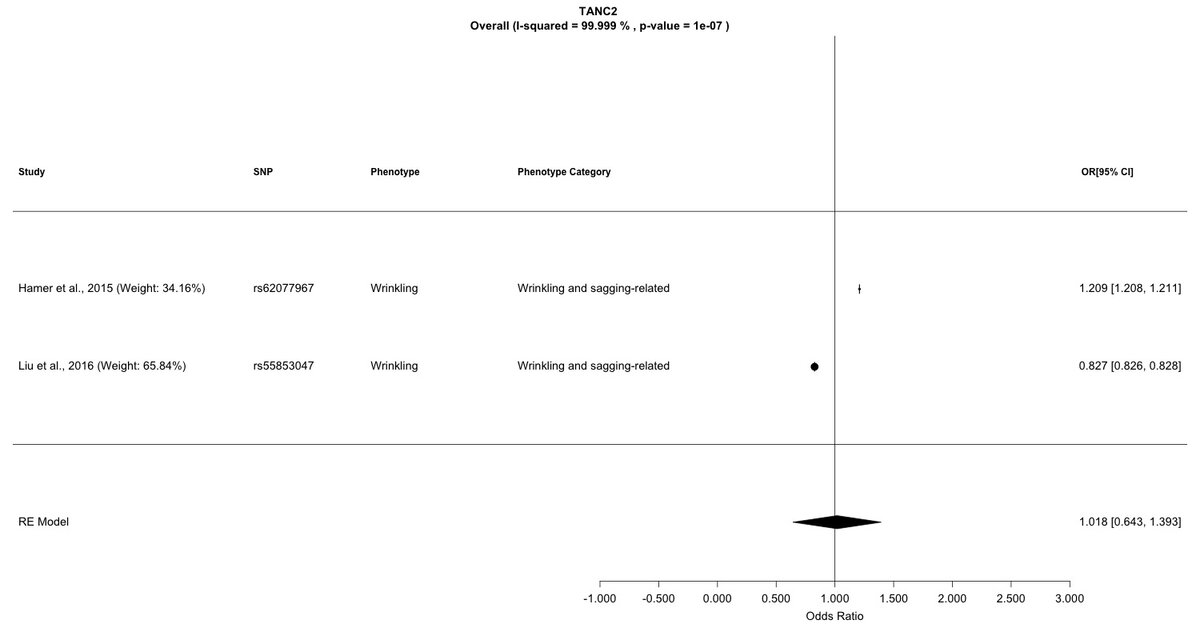

Supplement: Supplementary file 41 — Additional file 41. Forest plot of TANC2. [file 40101_2025_384_MOESM41_ESM.png]
